# Effects of high-definition tDCS targeting individual motor hotspot with EMG-driven robotic hand training on upper extremity motor function: a pilot randomized controlled trial

**DOI:** 10.1186/s12984-024-01468-w

**Published:** 2024-09-20

**Authors:** Chengpeng Hu, Chun Hang Eden Ti, Kai Yuan, Cheng Chen, Ahsan Khan, Xiangqian Shi, Winnie Chiu-wing Chu, Raymond Kai-yu Tong

**Affiliations:** 1 Department of Biomedical Engineering, The Chinese University of Hong Kong, Hong Kong SAR, China; 2 Department of Imaging and Interventional Radiology, The Chinese University of Hong Kong, Hong Kong SAR, China

**Keywords:** EMG-driven robotic hand, High-definition transcranial direct current stimulation, Personalized stimulation montage, Stroke rehabilitation, Task-based fMRI, Upper extremity

## Abstract

**Background:**

Delivering HD-tDCS on individual motor hotspot with optimal electric fields could overcome challenges of stroke heterogeneity, potentially facilitating neural activation and improving motor function for stroke survivors. However, the intervention effect of this personalized HD-tDCS has not been explored on post-stroke motor recovery. In this study, we aim to evaluate whether targeting individual motor hotspot with HD-tDCS followed by EMG-driven robotic hand training could further facilitate the upper extremity motor function for chronic stroke survivors.

**Methods:**

In this pilot randomized controlled trial, eighteen chronic stroke survivors were randomly allocated into two groups. The HDtDCS-group (*n* = 8) received personalized HD-tDCS using task-based fMRI to guide the stimulation on individual motor hotspot. The Sham-group (*n* = 10) received only sham stimulation. Both groups underwent 20 sessions of training, each session began with 20 min of HD-tDCS and was then followed by 60 min of robotic hand training. Clinical scales (Fugl-meyer Upper Extremity scale, FMAUE; Modified Ashworth Scale, MAS), and neuroimaging modalities (fMRI and EEG-EMG) were conducted before, after intervention, and at 6-month follow-up. Two-way repeated measures analysis of variance was used to compare the training effect between HDtDCS- and Sham-group.

**Results:**

HDtDCS-group demonstrated significantly better motor improvement than the Sham-group in terms of greater changes of FMAUE scores (F = 6.5, *P* = 0.004) and MASf (F = 3.6, *P* = 0.038) immediately and 6 months after the 20-session intervention. The task-based fMRI activation significantly shifted to the ipsilesional motor area in the HDtDCS-group, and this activation pattern increasingly concentrated on the motor hotspot being stimulated 6 months after training within the HDtDCS-group, whereas the increased activation is not sustainable in the Sham-group. The neuroimaging results indicate that neural plastic changes of the HDtDCS-group were guided specifically and sustained as an add-on effect of the stimulation.

**Conclusions:**

Stimulating the individual motor hotspot before robotic hand training could further enhance brain activation in motor-related regions that promote better motor recovery for chronic stroke.

**Trial registration:**

This study was retrospectively registered in ClinicalTrials.gov (ID NCT05638464).

**Supplementary Information:**

The online version contains supplementary material available at 10.1186/s12984-024-01468-w.

## Introduction

Stroke causes considerable deterioration of upper extremity (UE) sensorimotor function [1]. Given the complexity of hand functions, rehabilitation aiming at restoring hand function remains a major challenge [2]. Transcranial direct current stimulation (tDCS) can modulate cortical excitability and interhemispheric balance [3–6] via long-term potentiation (LTP) and long-term depression (LTD) of the stimulated neuronal pools [7, 8]. Previous studies concluded that tDCS as adjunctive therapy in UE motor recovery was promising [9–13]. However, conventional tDCS which uses rubber pad electrodes (typically 35 cm^2^ ) stimulating a large area of the cortex might not explicitly target the motor activity of interest. The lack of focality and specificity of tDCS might induce inconsistent stimulation effects on stroke rehabilitation [14–17], especially with the heterogeneous lesion profiles and cortical function reorganization within stroke individuals. Our recent studies also found that conventional tDCS delivered varied electric field (EF) magnitudes on individual primary motor cortex (M1) for stroke survivors, in which the lesional profiles could influence current flow and the EF distribution [18], and this EF variation affected stimulation effect on resting-state functional connectivity, where subjects with higher EF strength exhibited a greater increase in functional connectivity after stimulation [19].

Recently, high-definition tDCS (HD-tDCS) was developed to increase the spatial focality of current by using small surface area of electrodes (less than 2 cm diameter) [20]. The arrays of 4 × 1 ring electrode configuration on the targeted cortex allow the generation of focal EF patterns, which contributed to more efficient induction of neuroplasticity than conventional tDCS [21]. Another challenge is how we identify the specific target for HD-tDCS stimulation. Especially for stroke survivors, the individual anatomical variation of the lesion profiles drastically impacts the current flow [22]. In addition, brain activation is often reorganized in cortical areas inter- or intra-hemispheres distant from the lesions [23, 24]. As indicated by task-based fMRI, the motor hotspot shifted from the primary motor cortex (M1) to the perilesional secondary motor regions after stroke. For example, the premotor cortex (PMC) and supplementary motor area (SMA) play crucial roles in coordinating and compensating the functions for M1 deficiency [25, 26]. Consequently, using a conventional one-montage-fit-all tDCS approach, in which the anode is only placed above the ipsilesional M1, may not precisely stimulate the reorganized brain. It was suggested that for stroke survivors with cortical reorganization at a network level, designing HD-tDCS montages that target multiple sensorimotor hotspots could be more beneficial than stimulating M1 alone [27].

The personalization of tDCS montages that involves multiple individual-specific stimulation targets can overcome the issues of specificity and focality in conventional tDCS. A recently proposed montage that utilized HD-tDCS targeting multiple regions was found to modulate corticospinal excitability twice the magnitude of conventional tDCS on healthy subjects [27]. The placements of the electrodes were determined by best matching the EF generated from multiple electrodes to the spatial topography of the individual M1 resting-state functional connectivity derived from resting-state fMRI. Based on this approach, the optimization strategies for realizing personalized HD-tDCS were studied for stroke, including utilizing task-based fMRI to identify the individual somatosensory and motor representations [28, 29], and using finite element modeling (FEM) to account for anatomical features and various lesion profiles due to the heterogeneity of stroke [30]. However, the long-term intervention effectiveness of personalized HD-tDCS has not yet been studied in chronic survivors. Investigating the intervention effect would provide valuable insights into personalized stimulation protocols for stroke rehabilitation.

This study aimed to explore the add-on effect of the stimulation by designing personalized HD-tDCS followed by an EMG-driven robotic hand (EMG-RH) training protocol. EMG-RH uses surface EMG to record the user’s muscle contraction and control the robotic hand, allowing for power-assisted hand grasping and opening training driven by the user’s intention [31, 32]. The voluntary motor efforts, control, and proprioceptive feedback from EMG-RH enhance the integration of the central-peripheral neural circuits [33]. Although it was suggested to apply brain stimulation simultaneously with other therapies, which underlined the ‘activity-selectivity’ effect of tDCS [34]. Previous studies revealed the improvement of motor performance may be greater when tDCS was applied immediately before robotic hand intervention than during or post protocols [35]. We delivered HD-tDCS before EMG-RH by avoiding the potential interference between stimulation and intention-driven process during EMG-RH training. It was reported that tDCS has neuromodulatory after-effects around 30–40 min [36]. Our previous study also reported that HD-tDCS could modulate cortico-muscular integration for more than 40 min [37]. By applying HD-tDCS before EMG-RH training, the elevated motor network excitability could further facilitate the integration of central-peripheral neural circuits.

We hypothesized that HD-tDCS targeting individual ipsilesional motor hotspot would promote adaptive neuroplasticity that consolidates motor relearning process when combined with EMG-RH training. To verify our hypothesis, we examined motor recovery by clinical scores immediately and 6 months after training. A thorough investigation of the neural plasticity was performed with multimodal neuroimaging techniques, with task-based fMRI studying the interhemispheric activation, and with EEG/EMG focusing on the central-peripheral synchrony. To test the specificity of the HD-tDCS, we compared the overlapped regions between the optimized EFs and hand-task brain activation immediately and 6 months after training.

## Methods

### Study design

This is a pilot double-blinded randomized controlled trial with a 6-month follow-up. This study aimed to explore the add-on intervention effects of personalized HD-tDCS in addition to EMG-RH training on UE motor function and neuroplasticity. Two groups were designed in this study, including the HDtDCS-group receiving personalized HD-tDCS with EMG-RH training, and the Sham-group receiving sham stimulation with EMG-RH training. Each participant received 20 sessions of intervention, with an average of 1–3 sessions per week. Each session began with 20 min of HD-tDCS or Sham stimulation, followed by 60 min of EMG-RH training. The assessments included clinical scales, MRI scanning, EEG-EMG, and EMG to evaluate the motor function improvement and the potential neuromodulation effects. The fMRI assessment was conducted at three timepoints, including *Pre* (within 3 days before intervention), *Post* (within 3 days immediately after 20 sessions of intervention), and *6 m Follow-up* (within 3 days at 6 months after the intervention). In addition to fMRI, the EEG-EMG, clinical scales, and EMG assessments were also performed at these three timepoints, and these assessments were arranged on different days from fMRI but still within the 3-day window. The recruitment, assessments, intervention, and follow-up were conducted in Hong Kong between 2021 and 2022. This study was approved by the Joint Chinese University of Hong Kong-New Territories East Cluster Clinical Research Ethics Committee (No. 2018.661). This study was registered with an identifier NCT05638464.

### Participants

The recruited subjects met the following inclusion criteria: (1) first-ever stroke, the duration after stroke exceeds 6 months; (2) mild to moderate UE motor function deficit, with Fugl-Meyer assessment of upper extremity (FMAUE) scores between 15 and 53 [38]; (3) detectable voluntary EMG signal from flexor digitorum (FD) and extensor digitorum (ED); (4) scored below 4 in the Modified Ashworth Score (MAS) of FD and ED; (5) sufficient cognitive function to follow instructions, with Mini-Mental State Examination scores more than 21. (6) no experience with robotic hand training, tDCS, HD-tDCS, or transcranial magnetic stimulation before. The participants were excluded with a history of epilepsy or any other contradictions of tDCS and MRI scans. All participants gave their written informed consent before the experiments. This study was conducted under the principles of the Declaration of Helsinki.

The expected benefits and risk of the recently developed personalized HD-tDCS with robotic hand training on chronic stroke patients are not available in previous studies. Taking into consideration ethical concerns and available resources [39], we estimated the sample size based on our recent EMG-driven robotic hand training, the mean expected improvement in FMAUE was around 3.31 with a standard deviation of 3.79 points [40]. The mean expected improvement after tDCS with robotic arm training was referred to as 8.73 points from work by Triccas et al. [41] with a similar group sample size (around 10 subjects in a group). A power calculation with *P* = 0.05 and 80% power suggested that at least 8 subjects per group would detect a significant difference between the two groups in terms of FMAUE. In this pilot randomized controlled trial, we screened 60 subjects from local community and enrolled 19 subjects.

Standard envelope randomization was utilized to ensure the unbiased 1:1 ratio allocation of subjects to two groups. Before the trial, the randomization sequence was computer generated by a research team member (who did not participate in group assignment of enrolled participants), and opaque envelopes containing two different colored cards were sealed following this sequence. At the start of the trial, the envelopes were opened one by one in a predetermined order by the enrolled subjects following the instructions of investigator. The color of the card that each subject chose indicated the group to which they were allocated for the study. Subjects and the outcome raters were blinded to the allocation.

**Fig. 1:**
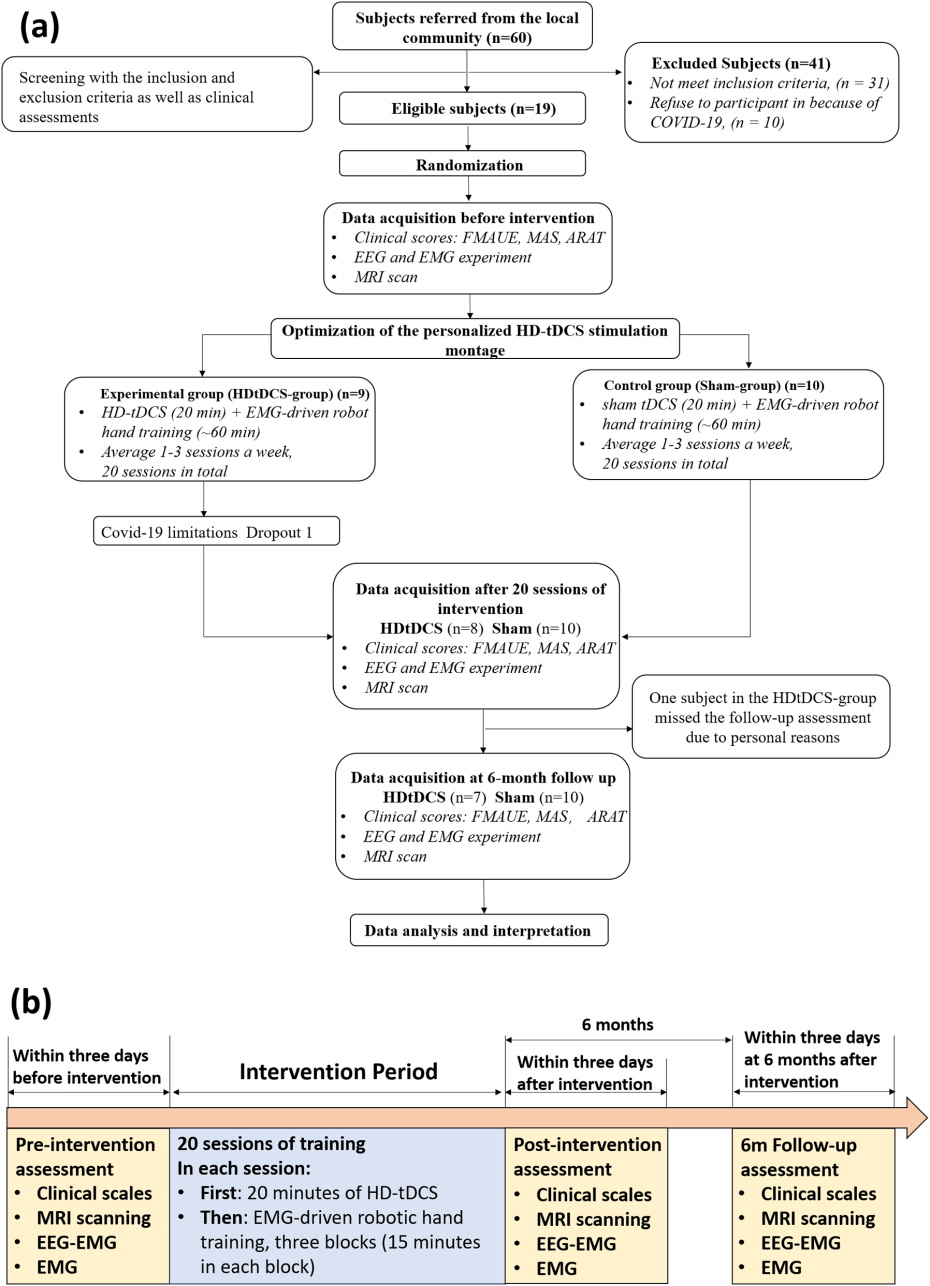
Flow chart of this study. (**a**). Flowchart of this pilot randomized controlled trial. (**b**). Workflow of this intervention study, including timelines of assessments and intervention. *Abbreviation* *HDtDCS-group* High-definition transcranial direct stimulation with EMG-driven robotic hand group; *Sham-group* sham stimulation with EMG-driven robotic hand group

Nineteen chronic stroke subjects were randomly allocated to HDtDCS-group (*n* = 9) and Sham-group (*n* = 10). The baseline demographics and clinical scores are demonstrated in Table 1. One subject in the HDtDCS-group dropped out because of COVID-19 restrictions. One subject from HDtDCS-group missed the *6 m Follow-up* assessment because of personal reasons. Figure 1 (a) shows the flowchart of this study. Figure 1 (b) shows the workflow of the assessments and the intervention.

**Table 1:** Clinical demographic at baseline and training information

Measures	HDtDCS-group (*n* = 9)	Sham-group (*n* = 10)	*P* value
Age (years)	56.0 ± 9.7	62.1 ± 10.8	0.216
Chronicity (month)	43.7 ± 40.6	62.2 ± 51.7	0.401
Gender (Female/Male)	7 / 2	5 / 5	0.463
Affected side (Right/left)	5 / 4	6 / 4	0.845
Stroke Type (Ischemia/Hemorrhagic)	6 / 3	7 / 3	0.876
Lesion site (Cortico-subcortical/Subcortical)	3 / 6	5 / 5	0.650
Lesion volume (cm^3^)	9.8 ± 15.3	13.1 ± 13.5	0.630
FMAUE	40.0 ± 6.4	40.5 ± 11.6	0.910
MAS of wrist	1.71 ± 1.14	1.66 ± 0.74	0.908
MAS of finger	1.80 ± 1.02	1.74 ± 0.93	0.895
ARAT	28.0 ± 14.1	29.0 ± 14.5	0.881
Minor side-effect incidence (itching, burning, or tingling)	5	4	0.637
Duration between Pre and Post assessment (days)	71.8 ± 30.0*	79.4 ± 18.8	0.517
Frequency of conventional therapy between *Post* and *Follow-up* (/week)	0.86 ± 1.07^#^	0.80 ± 0.92	0.908

Mean ± standard deviation was reported. No significant difference between the two groups was observed for all baseline clinical demographics and training information. *: Information from 8 subjects who finished all training sessions. ^#^: Information from 7 subjects who received follow-up assessment

*Abbreviations* *HDtDCS-group* High-definition transcranial direct stimulation with EMG-driven robotic hand group; *Sham-group* sham stimulation with EMG-driven robotic hand group; *FMAUE* Fugl-Meyer motor function assessment of upper extremity; *MAS* Modified Ashworth Scale; *Pre* before training; *Post* immediately after the training; *6 m Follow-up* six-month follow-up

### MRI data acquisition and processing

MRI data was acquired before intervention MRI scans were acquired using a 3T Siemens Prisma MRI scanner (Siemens Healthcare, Erlangen, Germany) with an 8-channel head coil, including T1-weighted anatomical images (TR/TE = 1900/2.93ms, flip angle = 9°, 176 slices, voxel size = $\:0.9\times\:0.9\times\:1.0\:{mm}^{3}$) using a T1-MPRAGE, and BOLD fMRI images ( TR/TE = 1200/30 ms, flip angle = 68°, 48 slices, voxel size = $\:3.0\times\:3.0\times\:3.0\:{mm}^{3}$) using an EPI-FID sequence. The sequences for task-based fMRI (tb-fMRI) were displayed using EPrime 3.0 (Psychology Software Tools, PA USA). Motor execution (ME) tasks were designed based on the EMG-RH training. During the ME task, two tennis balls were placed in the subject’s left hand and right hand respectively in advance. In case the ball fell out of the affected hand, adhesive tape was used to fix the tennis ball in the affected hand. Subjects were asked to grasp the corresponding hand when a mark of “left hand” or “right hand” appeared on the screen and were asked to maintain 6 s until a “rest” mark appeared. An event-related design was adopted with a randomized inter-trial interval ranging from 12 to 20 s. A total of 20 ME tasks including 10 using the affected hand and 10 using the unaffected hand were randomized adopted during the scanning, and it took around 7 min for tb-fMRI scanning [42]. The MRI scanning was performed for each subject at *Pre*, *Post*, and *6 m Follow-up* assessments.

The details of data processing are illustrated in Supplementary 1.1. As a result of the data processing, the *t-maps* during affected hand tasks were generated at *Pre*,* Post*,* and 6 m Follow-up* to evaluate cortical activation [43]. The tb-fMRI data of one subject in Sham-group was excluded from the analysis because of excessive motion artifacts. In addition, the MRI data acquired before intervention were also used to generate personalized HD-tDCS montages. Specifically, the *t-maps* acquired at *Pre* were recognized as the individual motor hotspot allocation, and the structural MRI acquired at *Pre* was used to determine individual lesion profiles and brain structure information.

### Personalized stimulation montage for multisite-HD-tDCS

**Fig. 2:**
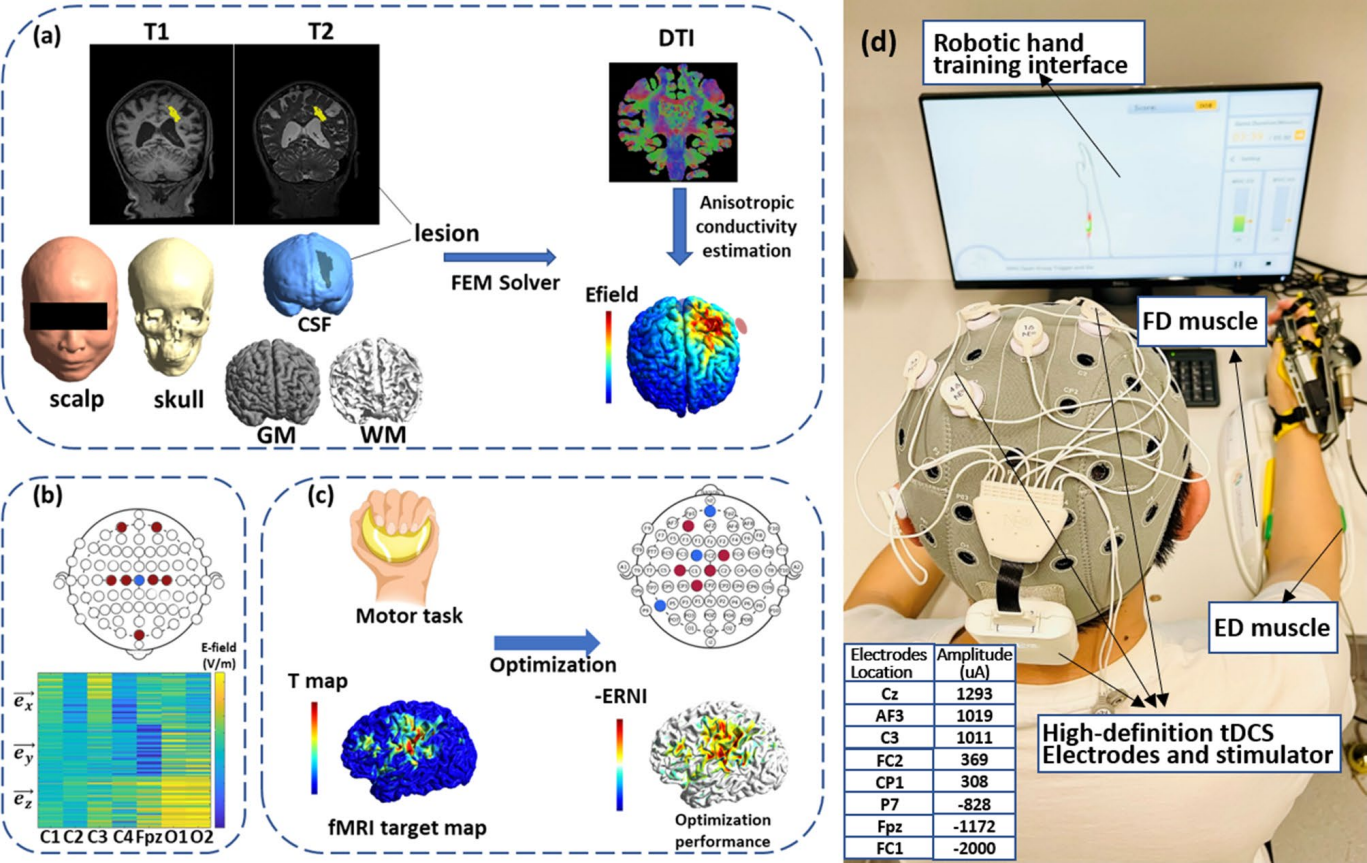
Optimization procedures and training protocol. (**a**) Generation of individualized finite element model. Segmentation of tissues was obtained from high-resolution structural T1 and T2 images and converted into volume conductor models consisting of six compartments. Simulation of the electric field was performed by solving the Laplace Equation using FEM solver after placing modeled electrodes at the desired locations. (**b**) Generation of leadfield matrix. Leadfield was generated by performing simulations with bipolar configurations, with anodes (red) placed at a defined set of locations (39 in total) and Cz (blue) as the cathode. The figure showed seven channels for illustration purposes. The column of the matrix represents the electric field in $\:\overrightarrow{{e}_{x}},\:\overrightarrow{{e}_{y}},\overrightarrow{{e}_{z}}$ direction over the volume conductor models. (**c**) Optimization of HD-tDCS montages. Stimulation targets were defined using individual fMRI activations during paretic motor tasks. Optimization was based on a distributed constrained maximum intensity method, which minimizes the Error Relative to No Intervention (ERNI). A value of higher -ERNI (red) indicates a smaller difference between the simulated and desired target electric field. (**d**) The stimulation montage of one subject was generated with the amplitude and location of each electrode. After 20 s of active/sham stimulation, the subject accepted robotic hand training. The active-assisted opening and grasping hand movements were cued by the instructions on the screen in front of the subject and triggered by the muscle contraction of the Extensor digitorum (ED) and Flexor digitorum (FD) respectively

The personalized stimulation montage was generated using MRI data for each subject from both HDtDCS-group and Sham-group. Details of optimization can be found in Supplementary 1.2. Briefly, a FEM was generated from individual structural T1 and T2 images (Fig. 2a), including six compartments (scalp, skull, cerebrospinal fluid, grey matter, white matter, and stroke lesions). Each compartment was assigned corresponding isotropic electrical conductivity values [44, 45]. Optimization of montages was then performed on individual FEM models following the procedures described in previous studies [46, 47]. Specifically, the individual fMRI t-map was used as target map to guide the EF distribution and generate the best-match montages (Fig. 2c). The Error Relative to No Intervention (ERNI) value was calculated to evaluate the optimization performance. A negative ERNI represents EFs approaching the target maps, indicating a better fit. Montages were set with the following constraints: (1) The number of electrodes is less than or equal to 8. (2) The total current inside the brain does not exceed 4 mA. (3) The current of each electrode does not exceed 2 mA. (4) The sum of the total current equals zero. The results of optimization were quantified by the total ERNI and the weighted cross-correlation (WCC) between the target EF and simulated EF, using the definition in a previous study [46]. The targeting Index and Miss-hit Index were calculated to show the stimulation details. The Targeting Index reveals the proportions of the regions in the ipsilesional activation map that were stimulated; the Miss-hit Index means the percentage of unactivated regions that were stimulated. The optimization generated the individual stimulation montage, including the location and current intensity of each electrode (Supplementary Table 2.1).

### Personalized HD-tDCS montages

**Fig. 3:**
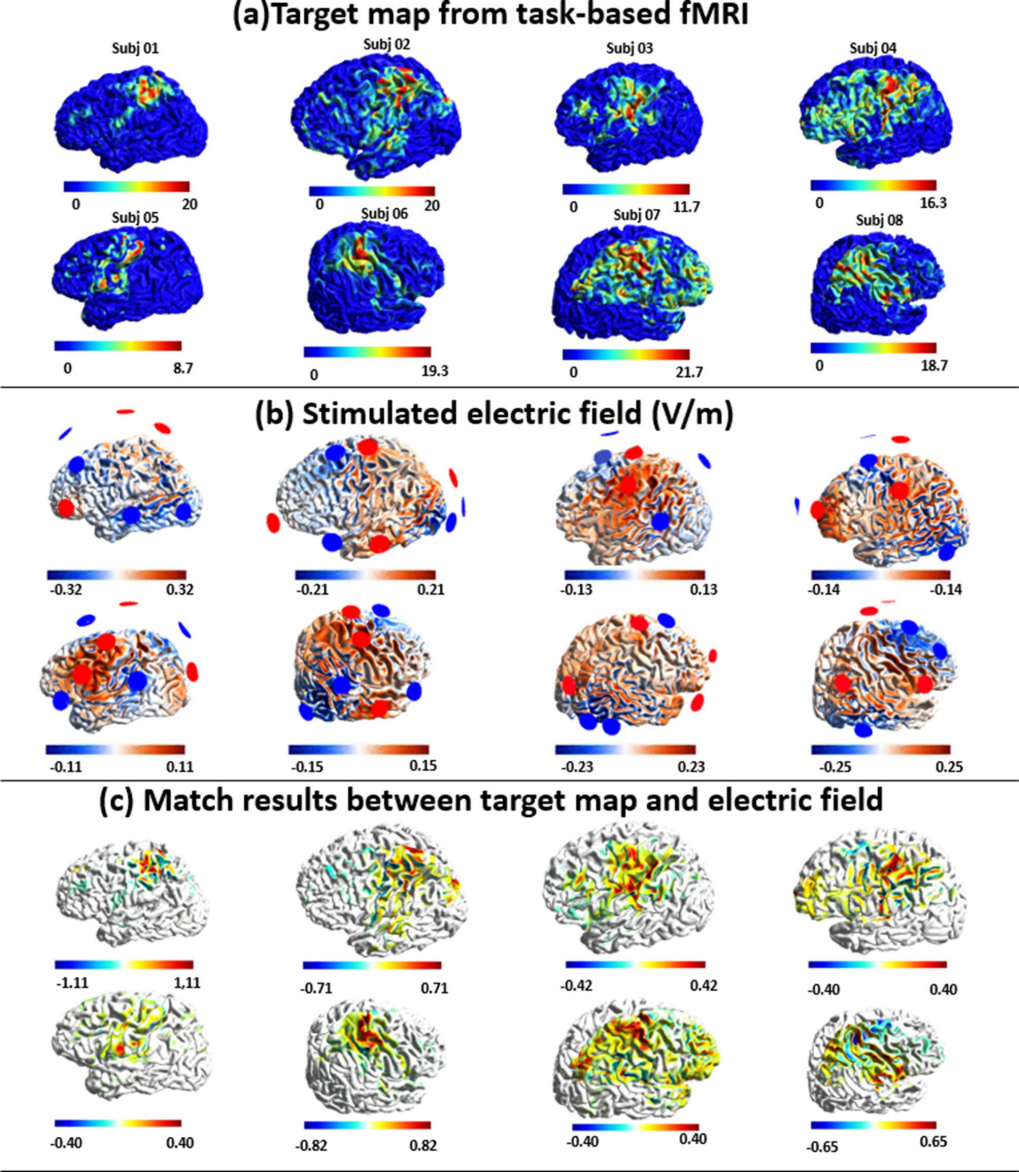
Optimization results of personalized high-definition-tDCS montages. Electric field simulation results using the optimized montages of eight chronic stroke subjects from the HDtDCS-group. (**a**) Individual target map from task-based fMRI. The map was generated from the grasping hand task-fMRI activation map. Individual hotspot locations can be found in Table 2. (**b**) Stimulated electric field. Normal EF component for optimized stimulation montages. Electrodes in red and blue represent the anode and cathode respectively, details of the individual montage information can be found in Supplementary Table 2.1. (**c**) Match results between target map and electric field. The optimization performance for each subject was quantified by Error Relative To No Intervention (ERNI). Positive values (red) indicate a better fit than no intervention, and negative values (blue) mean a worse fit than no intervention

**Fig. 4:**
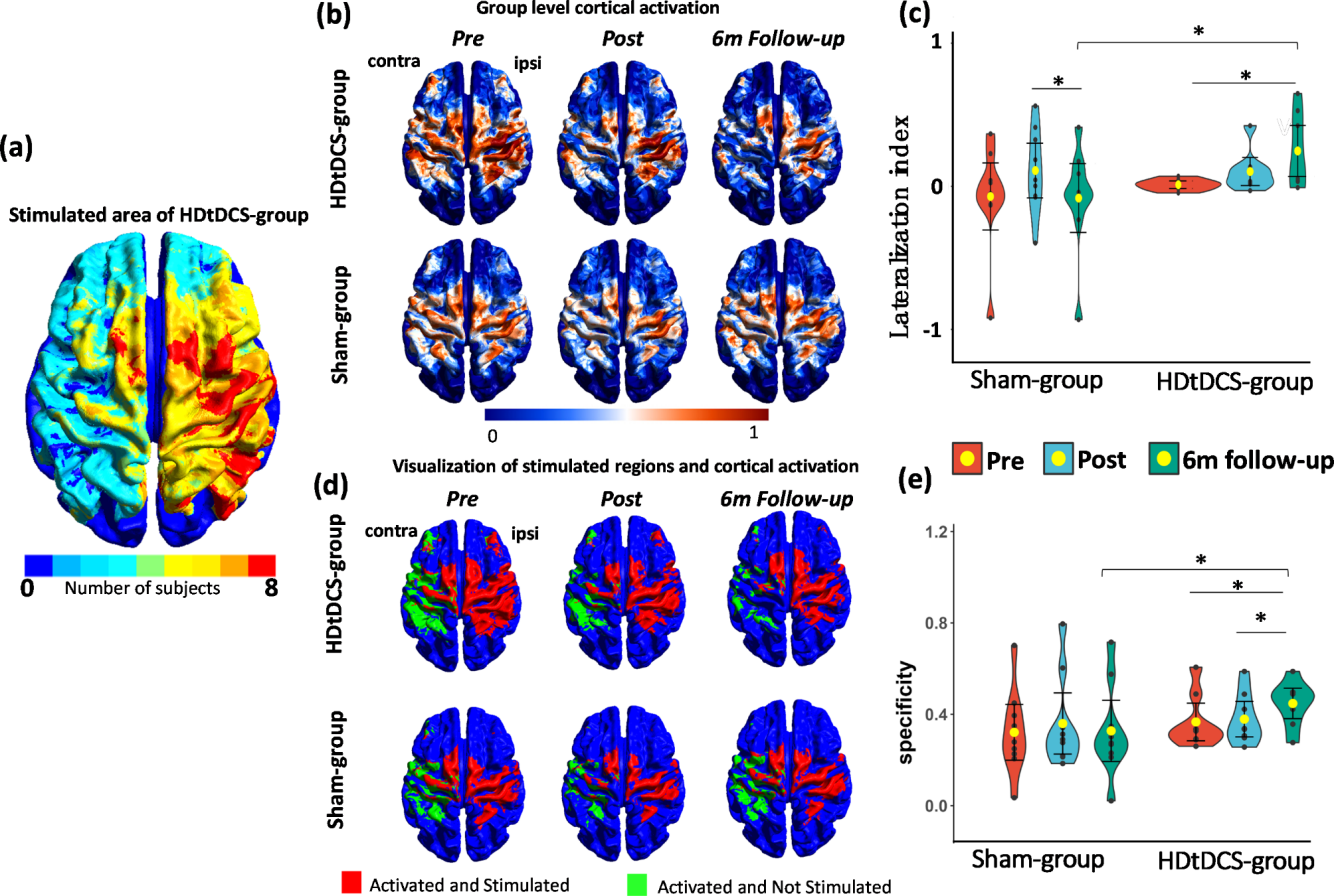
FMRI results. (**a**) Group level of the overlapped stimulated area of subjects from the HD-tDCS group, regions in red represent the area that all the subjects from the HDtDCS-group were stimulated. (**b**) Group level motor activation at *Pre*, *Post*,* and 6-month Follow-up* sessions. The activation maps were generated from the HDtDCS-group and Sham-group during grasping the paretic hand. (**c**) Violin plots with mean and standard deviation showing the comparison of laterality index for the HDtDCS-group and Sham-group at *Pre*, *Post*,* and 6-month Follow-up*. (**d**) The demonstration of the overlaps between activated and stimulated regions (red), and the activated regions that were not stimulated (green) at *Pre*, *Post*, *and 6-month Follow-up* sessions. Activations were focused on the stimulated regions and reduced in the non-stimulated regions *after intervention*, *and continuously at 6-month follow-up* for HDtDCS-group, indicating an enhanced Specificity of stimulation. (**e**) The violin plots with mean and standard deviation show the comparison of specificity in both groups at *Pre*, *Post*, *and 6-month Follow-up*. *Abbreviation* *HDtDCS-group* High-definition transcranial direct stimulation with EMG-driven robotic hand group; *Sham-group* sham stimulation with EMG-driven robotic hand group; *contra* contralesional hemisphere; *ipsi* ipsilesional hemisphere; *Pre* before training; *Post* immediately after the training; *6 m Follow-up* six-month follow-up. *: *P* < 0.05. The yellow dot and black bars represent the mean and standard deviation of the corresponding group

The optimization results for subjects from HDtDCS-group were demonstrated in Fig. 3. The reorganized brain activation varied across individuals (Fig. 3a), including the M1, SMA, ventrolateral and dorsolateral PMC, and superior and inferior parietal cortex. The location in the MNI space of individual hotspot was summarized, with an average shifted distance of 18.5 mm from the standard primary motor cortex (Table 2). The individual lesion profiles were demonstrated in Supplementary Table 2.2. Figure 3b shows the optimized individual EF using the generated montage. Figure 3c demonstrates the individual local ERNI maps to show the performance of the optimization, where the maps depicted focal stimulation on the targets with promising matching performance. An average value of $\:-685$ and 0.215 was achieved for ERNI and WCC respectively, which yielded comparable results with other optimization simulation studies [28]. Figure 4a shows the group-level overlapped electric field of HDtDCS-group, which indicates the M1, SMA, PMC, and inferior parietal cortex were targeted for all subjects in HDtDCS-group.

**Table 2:** Information of individual motor hotspot and optimization performance for HDtDCS-group

Subject in HDtDCS group	Hotspot Location			Shifted distance (mm)	Targeting Index (Overlap/IpsiActiv.)	Miss-hit Index (NonActiv./EF)	ERNI	WCC
	MNI_x	MNI_y	MNI_z					
1	-3.12	-16.39	52.62	35.49	0.6228	0.3200	-852	0.235
2	-51.65	-25.27	50.65	15.02	0.5159	0.3473	-90.5	0.149
3	-38.01	-25.12	44.80	11.62	0.6409	0.4391	-591	0.182
4	-29.08	-29.48	70.89	18.90	0.5219	0.1917	-688	0.231
5	-42.17	-23.11	50.02	7.37	0.7009	0.4600	-481	0.171
6	38.34	-26.76	54.20	5.10	0.5759	0.3423	-370	0.191
7	48.31	-20.00	35.65	22.90	0.5421	0.1749	-577	0.191
8	8.12	-11.69	57.06	31.63	0.4925	0.3512	-1830	0.370
mean	-	-	-	18.50	0.5766	0.3283	-685	0.215

*Abbreviations* *ERNI* Error Relative to No Intervention value; *WCC* Weighted Correlation Coefficient. The Targeting Index means the proportions of the regions of the ipsilesional activation area given stimulation. The miss-hit Index represents the proportions of the non-activated area that was stimulated

### HD-tDCS combined EMG-RH training

After we optimized the montage for the personalized HD-tDCS, we applied it to the intervention. During the intervention, both groups underwent 20 sessions of training, each session began with 20 min of HD-tDCS (Personalized HD-tDCS/Sham) and was then followed by 60 min of robotic hand training. An average of 1–3 sessions of training were delivered each week, and the average and standard deviation of training duration was 71.8 ± 30.0 days for the HDtDCS-group and 79.4 ± 18.8 days for Sham-group. The cost of the MRI scan for building stimulation montage was around USD 500 for each subject.

In this study, a brain electrical stimulation device (StarStim, Neuroelectrics, Barcelona, Spain) and a robotic hand (Hand of Hope, Rehab-Robotics, Hong Kong) were applied. In each training session, HD-tDCS was conducted before EMG-RH training, where subjects received 20-minute personalized stimulation with the optimized stimulation montages, including a 1-minute ramp-up and ramp-down period. For the Sham-group, only ramp-up and ramp-down stimulation was applied.

After HD-tDCS, EMG-RH training was conducted for both groups. In each session, EMG-RH training involved three blocks with 15 min of training in each block. Between two blocks, subjects took 5 min of rest. During EMG-RH, the EMG signals collected from voluntary contraction of the FD and ED muscles were used to trigger the active powered assistance for grasping and opening of the robotic hand, respectively. Movements were triggered when the EMG level exceeded pre-set threshold (10% of the Maximal Voluntary Contraction (MVC) measured before each session), at which our chronic stroke participants could comfortably and consistently trigger the activation of the robotic hand with their residual EMG during voluntary contraction. The threshold can be adjusted based on the participant’s performance and feedback to meet the best intervention effect adjusted by our experienced staff. Each power-assisted movement would take 5 s to complete. During the assistive training, subjects were instructed to keep contracting muscles until the robot hand stopped, and then perform the next movement following the instruction on the screen (Fig. 2d). The repetitions of both grasping- and opening-hand were between 100 and 180 times for each session. The details of the intervention protocol are demonstrated in Supplementary 1.3.

### Outcome measures

To evaluate the intervention effects on upper extremity motor function, the primary outcome was the FMAUE which measures motor skill, coordination, and speed of the UE. FMAUE consists of 33 items, each of which adopts a 3-point scoring system from 0 to 2 points, with a total score of 66 points [48]. The secondary outcomes included MAS for finger (MASf) and wrist (MASw), Action Research Arm Test (ARAT), and neuroimaging measures including tb-fMRI, EEG-EMG, and EMG assessments. Clinical assessments were conducted by a licensed physical therapist who was blinded from the training procedure and other evaluations.

### Task-based fMRI analysis

To explore the interhemispheric activation pattern after the intervention, lateralization index ($\:LI$) during the ME tasks at *Pre*,* Post*, and *6 m Follow-up* were computed. $\:LI$ refers to the normalized difference between the number of activated voxels in the ipsilesional and contralesional hemispheres. The voxels located in the sensorimotor areas (motor, premotor, and somatosensory regions) were masked for the $\:LI$ calculation [49]. The $\:LI$ value was computed using formula (1).

1 $$\:LI=\:\:\frac{{N}_{ipsi}\:-\:{N}_{contra}}{{N}_{ipsi}\:+\:{N}_{contra}}$$

where $\:{N}_{ipsi}$ stands for the number of activated voxels in the ipsilesional hemisphere. $\:{N}_{contra}$stands for the number of activated voxels in the contralesional hemisphere. Therefore, the range of $\:LI$ value was between − 1 and 1. A LI value of 1 represents the activation lies purely in the ipsilesional hemisphere and − 1 represents the activation purely relies on the contralesional hemisphere.

To investigate how the stimulation affected the activation patterns after training, the Specificity was calculated to quantify the specificity of the HD-tDCS by using the simulated EFs and the tb-fMRI activation maps at *Pre*, *Post*, and *6 m Follow-up* according to formula (2).

2 $$\:{Specificity}_{ij}=\frac{2\left({N}_{\left({E}_{i}|{A}_{ij}\right)}\times\:{N}_{\left({A}_{ij}|{E}_{i}\right)}\right)}{{N}_{\left({E}_{i}|{A}_{ij}\right)}+{N}_{\left({A}_{ij}|{E}_{i}\right)}}$$

where subscripts *i* and *j* denote the individuals and evaluation sessions respectively. $\:{E}_{i}$ represents the electric field of subject *i*, $\:{A}_{ij}$ represents the activation mask of subject *i* in *j* evaluation session. $\:{N}_{\left(E|{A}_{ij}\right)}$ represents the the proportion of activated regions that are stimulated by tDCS, and $\:{N}_{\left({A}_{ij}|E\right)}$ represents the proportion of activation given the stimulated regions. Specificity value ranges from 0 to 1, and measures how well the simulated EFs and tb-fMRI align with each other. A value 1 representing a perfect overlap between the EF and activation map, and value 0 representing no overlapping. The calculation of $\:LI$ and Specificity were detaily illustrated in Supplementary 1.4.

### EEG-EMG measurement and cortico-muscular coherence (CMC)

To evaluate the connection between the central neural system and peripheral muscles during motor tasks, EEG-EMG assessments were conducted at *Pre*, *Post*, and *6 m Follow-up* assessments for each subject. During the data acquisition, the 128-channel Neuroscan amplifier (SynAmps2, Neuroscan Inc, Herndon, USA) was used to collect EEG and EMG signals, and a 128-channel Quik-Cap EEG cap was used. Two pairs of bipolar EMG electrodes were carefully placed over the affected FD and ED muscles. The EEG and EMG data were simultaneously collected from two motor tasks: isometric contraction of the grasping and opening paretic hand, each task lasted around 5 min, including 3 contraction trials with each trial lasting 40 s and two 1-minute intermediate breaks. Subjects were instructed to maintain a steady 30% MVC contraction. An online EMG feedback interface was shown in front of subjects to ensure muscle contraction stability (Supplementary Fig. 1.2).

After data acquisition, the time-aligned EEG and EMG signals were offline processed and CMC parameters were calculated (See details in Supplementary Methods 1.5). Cortico-muscular coherence (CMC) reflects the functional connection between cortical and muscles based on the spectral correlation between EEG and EMG signals [50]. The magnitude-squared coherence spectrum was calculated based on the power spectral density estimation with formula (3),

3 $$\:{C}_{XY}\left(F\right)=\frac{\left|\right|{P}_{XY}{\left(f\right)}^{2}}{{P}_{XX}\left(f\right){P}_{YY}\left(f\right)\:}$$

where $\:{P}_{XX}\left(f\right)$ and $\:{P}_{YY}\left(f\right)$ were the auto-power spectral density (PSD) of EEG and EMG signals (represented as *X* and *Y*) throughout segments for a given frequency *f*, and $\:{P}_{XY}\left(f\right)$ is the cross-power spectral density between them [51]. The CMC values range from 0 to 1, with higher values indicating stronger cortico-muscular interaction.

For chronic stroke survivors, the CMC-related motor function might shift away from the ipsilesional M1 area as reported in previous studies [52]. To mitigate this effect, a cluster of five channels (C4, FCC4H, FCC6H, CCP4H, and CCP6H) located at the primary motor cortex, was selected as the target region. The frequencies of interest were defined in Alpha band (8–13 Hz), Beta band (13–30 Hz), and low Gamma band (30–45 Hz). The CMC value was defined as the “Peak” coherence, namely the largest coherence in the given frequency band. The CMC topographies generated at the peak-CMC-relative frequency from selected channels were averaged in different frequency bands at three evaluation sessions. Two CMC parameters were computed, including CMC value of FD during grasping hand ($\:{CMC}_{FDgrasp}$) and CMC value of ED during opening hand ($\:{CMC}_{EDopen}$). The detailed description of EEG-EMG set-up, procedures, and preprocessing were demonstrated in Supplementary 1.5.

### EMG assessment

Muscle activation was measured using EMG. During assessments, participants were instructed to perform unassisted, repetitive, full-hand grasping and opening with a comfortable muscle contraction. At the same time, EMG signals were recorded from FD and ED. The co-contraction index (CI) was calculated during grasping and opening tasks between FD and ED as computed with formula (4):

4 $$\:{CI}_{k}=\:\frac{1}{T}{\int\:}_{0}^{T}{A}_{k}\left(t\right)dt$$

where $\:{A}_{k}$ was the overlapping activity of normalized EMG linear envelopes for the FD/ED muscle pair during the movement *k* (i.e. hand grasping and opening), and *T* was the length of the signal. The CI of FD/ED muscle varied from 0 (no overlapping of muscle contractions) to 1 (complete overlapping of two maximal muscle contractions with both EMG activation levels kept at 1 during relative movement). A higher CI value indicates enlarged co-contraction phase of two muscles, which leads to less energy-efficient movement, whereas a lower CI suggests improved muscle coordination [53, 54]. The details of EMG assessments are demonstrated in Supplementary 1.6.

### Statistical analysis

Statistical analysis was performed using SPSS 20 (IBM, Armonk, NY, USA). Violin plots with mean and standard deviation were used to demonstrate the variables. MASf and MASw were reported as sum of flexion and extension of fingers and wrist, respectively. Intention-to-treat analysis was used to handle the missing data. The Shapiro-Wilk test was used to check data distribution properties. The demographic and baseline characteristics were compared between two groups using t-test (or Mann-Whitney U test) or Fisher exact tests. For normally distributed datasets, two-way repeated measures analysis of variance (ANOVA) was performed to explore the effect of time (*Pre*, *Post*, and *6 m Follow-up*), group (HDtDCS-group and Sham-group), and time × group interaction. Then paired t test was used for within-group multiple comparison. Partial Eta Squared (*η*^2^) and Cohen’s d value were reported to demonstrate the effect size [55]. *η*^2^ greater than 0.138 represented a large effect. *η*^2^ greater than 0.059 represented a moderate effect, and *η*^2^ greater than 0.01 represented a small effect. For non-parametric datasets, the Friedman test was applied for repeated measurements and the Wilcoxon’s signed-rank test was used for multiple comparisons, in addition, the effect size (rank biserial correlation, *r*) was calculated from the *z*-value of the Wilcoxon signed-rank test. Within-group comparisons were performed for the outcomes, including *Pre* vs. *Post*, *Pre* vs. *6 m Follow-up*, and *Post* vs. *6 m Follow-up*. The alpha level for significance was set at *P* < 0.05. Bonferroni correction was used when investigating multiple within-group comparisons, resulting in *P* < 0.0166 as the significance threshold [56].

## Results

The comparison of baseline clinical scores and demographics showed no significant between-group difference (Table 1). No serious adverse effect (neurological deterioration) was reported from HDtDCS- or Sham-group. The minor side-effects occurred with similar incidence in both groups (*P* = 0.637). All minor adverse effects were fully reversible.

### Task-based fMRI

Significant time effect (*P* = 0.038) and time × group interaction effect were found for $\:LI$ values (F = 5.25, *P* = 0.011, *η*^2^: 0.258). Significant time effects was revealed in both HDtDCS-group (*P* = 0.025) and Sham-group (*P* = 0.037). Pairwise comparison demonstrated a significant increase of $\:LI$ in *6 m Follow-up* compared with *Pre* in HDtDCS-group (*P* = 0.016, Cohen’s d = 1.11), and a significant decrease of $\:LI$ was found in *6 m Follow-up* compared with *Post* in the Sham-group (*P* = 0.014, Cohen’s d = 1.049) (Fig. 4b and c). Analysis of Specificity showed significant time × group interaction (F = 5.14, *P* = 0.013, *η*^2^: 0.255). Figure 4d depicts the group-level overlapped regions between electric fields of the stimulated areas from both groups. After intervention, the activation of motor hotspot was more concentrated in the stimulated motor regions, especially in HDtDCS-group at the *6 m Follow-up*, while in Sham-group, the overlapped region proportion reduced, and activation shifted back to contralesional motor regions. Pairwise analysis identified a significant increase in Specificity at *6 m Follow-up* when compared with *Pre* (*P* = 0.013, Cohen’s d = 1.162) and compared with *Post* (*P* = 0.014, Cohen’s d = 1.143) in HDtDCS-group, while no significant change of Specificity was observed in the Sham-group (*Pre* vs. *Post*, *P* = 0.169; *Pre* vs. *6 m Follow-up*, *P* = 0.727; *Post* vs. *6 m Follow-upP* = 0.124). (Fig. 4e). These results suggest that at *6 m Follow-up*, individuals in HDtDCS-group relied more on the ipsilesional motor cortex to perform hand tasks compared to Sham-group. Meanwhile, the reorganized activation in motor regions concentrated on the area being stimulated, which was not found in Sham-group.

### Clinical scores

**Fig. 5:**
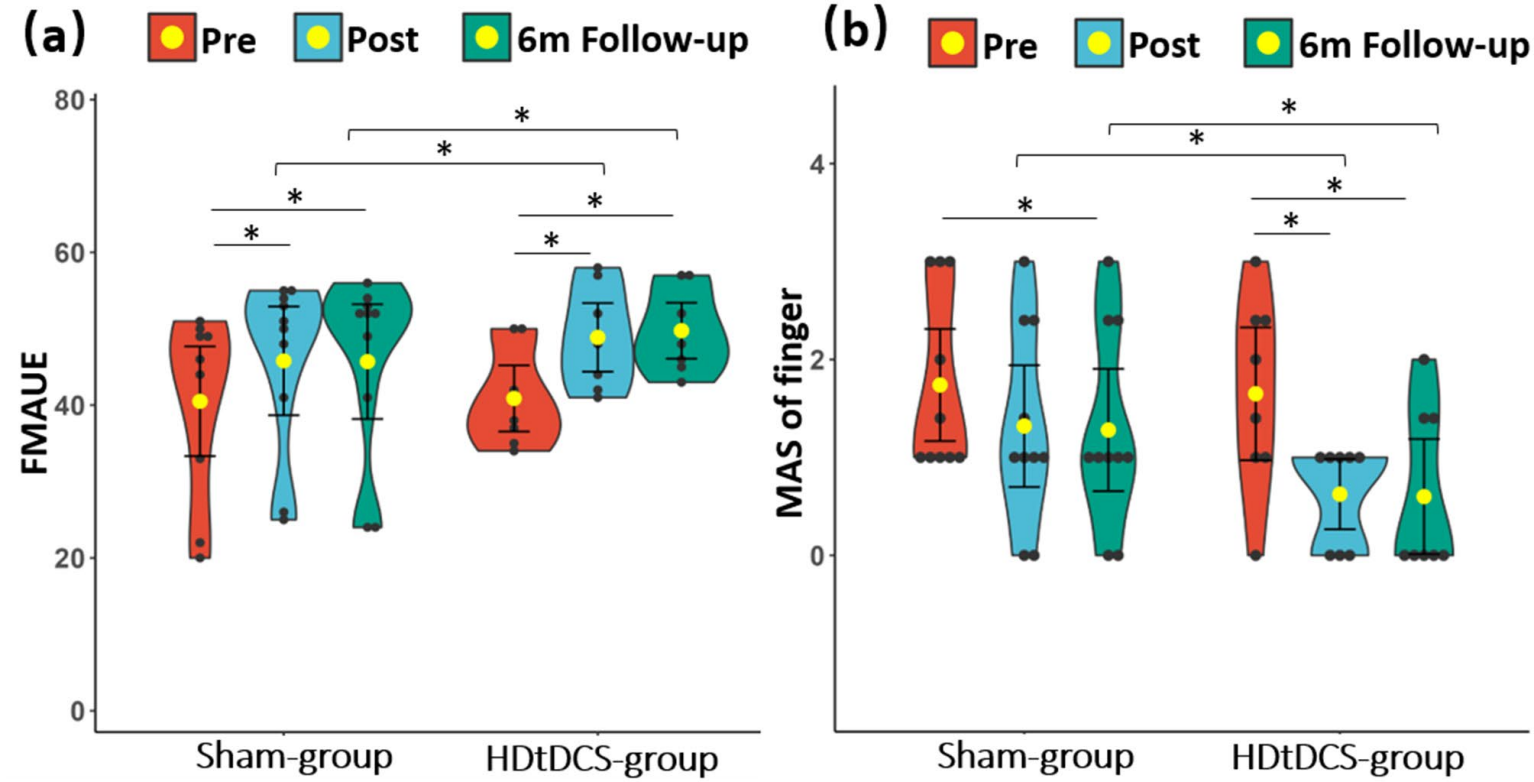
Clinical results. (**a**) Comparisons of FMAUE scores at *Pre*, *Post*, and *6 m Follow-up* sessions. (**b**) Comparisons of MAS of fingers at *Pre*, *Post*, and *6 m Follow-up* sessions. *Abbreviation* *FMAUE* Fugl-Meyer upper extremity motor scales; *MAS* modified Ashworsh scale; *HDtDCS-group* High-definition transcranial direct stimulation with EMG-driven robotic hand group; *Sham-group* sham stimulation with EMG-driven robotic hand group; *Pre* before training; *Post* immediately after the training; 6 m Follow-up, six-month follow-up. *: *P* < 0.05. The yellow dot and black bars represent the mean and standard deviation of the corresponding group

Both groups showed significant increases in FMAUE and ARAT, and decreases in MASw, and MASf over time (Ps < 0.016). The significant time × group interaction was found for FMAUE (F = 6.5, *P* = 0.004, *η*^2^ : 0.290) and MASf (F = 3.6, *P* = 0.038, *η*^2^: 0.185). HDtDCS-group presented greater increases in FMAUE (HDtDCS-group: *Pre* 40.9 ± 6.2, *Post*: 48.9 ± 6.5, *6 m Follow-up*: 49.8 ± 5.7; Sham-group: *Pre* 40.5 ± 11.6, *Post*: 45.8 ± 11.5, *6 m Follow-up*: 45.7 ± 12.1) at *Post* (between-group *P* = 0.034, Cohen’s d = 1.102) and *6 m Follow-up* (between-group *P* = 0.002, Cohen’s d = 1.733) compared with the Sham-group; as well as more decrease in MASf (HDtDCS-group: *Pre* 1.65 ± 0.98, *Post*: 0.62 ± 0.52, *6 m Follow-up*: 0.60 ± 0.85; Sham-group: *Pre* 1.74 ± 0.92, *Post*: 1.32 ± 1.00, *6 m Follow-up*: 1.28 ± 1.00) at *Post* (between-group *P* = 0.031, Cohen’s d = 1.125) and *6 m Follow-up* (between-group *P* = 0.023, Cohen’s d = 1.194) compared with the Sham-group (Fig. 5). No significant time × group interaction was observed for ARAT scores (F = 2.648, *P* = 0.086, *η*^2^ = 0.142), it demonstrated a similar improvement trend as the FMAUE scores but with no significant difference between groups. The increase in ARAT scores for HDtDCS-group was 7.88 ± 1.64 at *Post* (*P* < 0.001, Cohen’s d = 4.796) and 8.75 ± 3.06 at *6 m Follow-up* (*P* < 0.001, Cohen’s d = 2.860), while for Sham-group, it was 6.5 ± 2.17 (*P* < 0.001, Cohen’s d = 2.991) at *Post* and 6.10 ± 2.64 at *6 m Follow-up* (*P* < 0.001, Cohen’s d = 2.307). No between-group difference in ARAT was observed at Post (*P* = 0.158 Cohen’s d = 0.702) or at *6 m Follow-up* (*P* = 0.066 Cohen’s d = 0.935). Details of clinical scores comparison can be found in Supplementary Table 2.3. The results suggest that individuals in the HDtDCS-group gained more improvement in UE motor function, whilst also greater reduction in finger spasticity, compared to Sham-group.

### Cortico-muscular coherence (CMC) and EMG measures

**Fig. 6:**
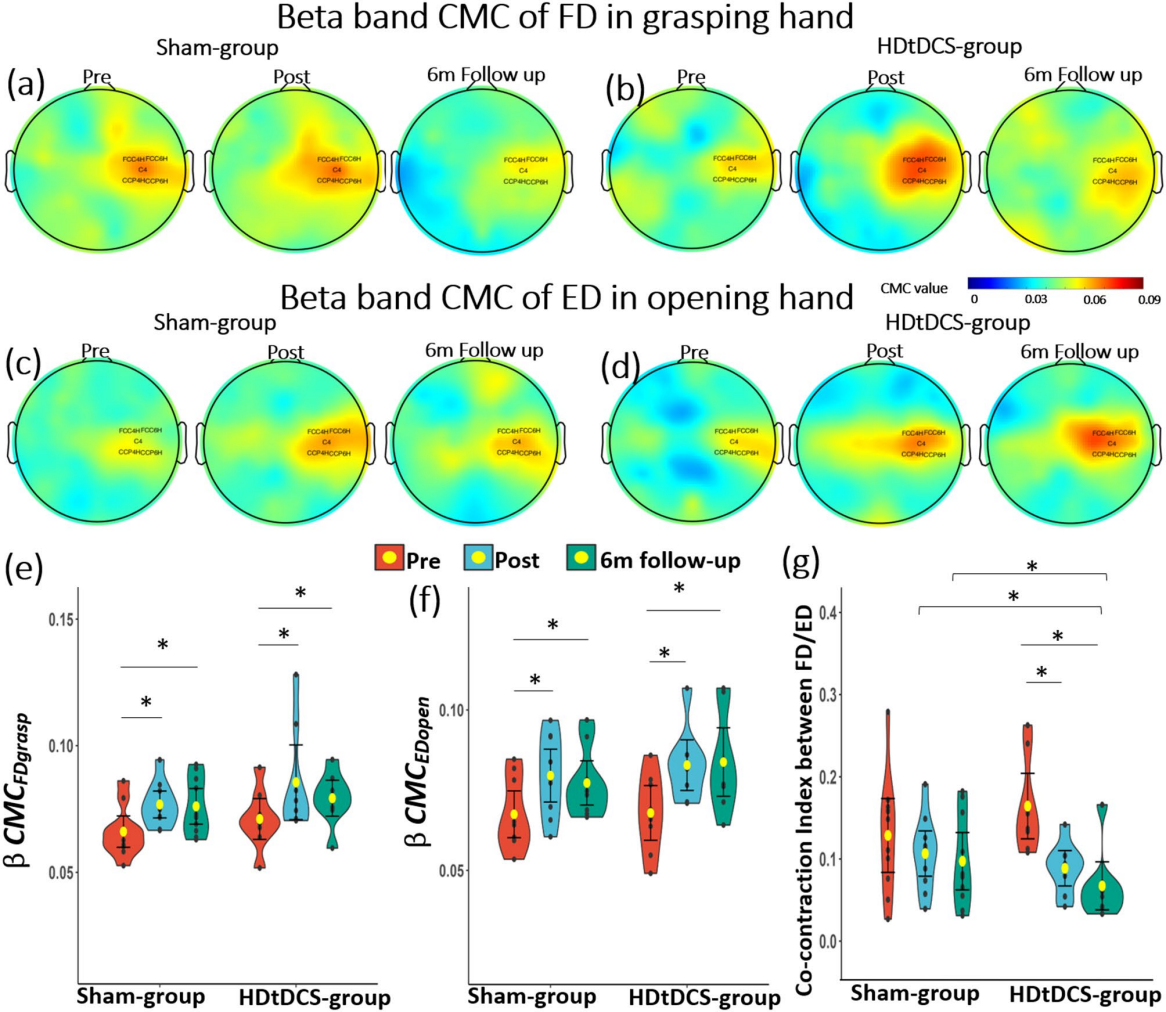
CMC and EMG results. Topography of mean CMC value from five channels (FCC4H, FCC6H, C4, CCP4H, CCP6H) around M1 during agonist muscle task in Beta band before, after training, and follow-up assessment. (**a**), Beta band CMC topography of Flexor digitorum during grasping hand of HDtDCS-group; (**b**), Beta band CMC topography of Flexor digitorum during grasping hand of Sham-group; (**c**), Beta band CMC topography of Extensor digitorum during opening hand of HDtDCS-group; (**d**), Beta band CMC topography of Extensor digitorum during opening hand of Sham-group; (**e**), Comparisons of Beta CMC of FD during grasping hand at *Pre*, *Post*,* and 6-month Follow-up*; (**f**), Comparisons of Beta CMC of ED during opening hand at *Pre*, *Post*,* and 6-month Follow-up*. (**g**) Comparisons of co-contraction index between FD and ED during opening hand at *Pre*, *Post and 6-month Follow-up*. *Abbreviation* *HDtDCS-group* High-definition transcranial direct stimulation with EMG-driven robotic hand group; *Sham-group* sham stimulation with EMG-driven robotic hand group; *CMC* cortico-muscular coherence; *Pre* before training; *Post* immediately after the training; 6 m Follow-up, six-month follow-up; *FD* flexor digitorum; *ED* extensor digitorum; *: *P* < 0.05

The agonist muscle tasks results showed a significant time effect for the *BetaCMC* with an increase in $\:{CMC}_{FDgrasp}$ (*P* < 0.001) and $\:{CMC}_{EDopen}$ (*P* < 0.001) (Fig. 6). Both groups showed a significant increase of $\:{BetaCMC}_{FDgrasp}$ and $\:{BetaCMC}_{EDopen}$ at *Post* and *6 m Follow-up* assessments (Ps < 0.016), while no significant time × group interaction was found. There were no significant changes in CMC variables in the Alpha and Gamma bands (Ps > 0.05). EMG measures indicated the significant time effect of $\:{CI}_{open}$ (*P* < 0.05). Significant time × group interaction was observed for $\:{CI}_{open}$ (F = 4.4, *P* = 0.02, *η*^2^: 0.216), and HDtDCS-group presented greater reductions in $\:{CI}_{open}$ value at *Post* (Between-group *P* = 0.014, Cohen’s d = 1.308) and *6 m Follow-up* (Between-group *P* = 0.035, Cohen’s d = 1.090) compared to Sham-group (Fig. 6g). Details of CMC and EMG parameters can be found in Supplementary Table 2.4 and Table 2.5. The CMC and EMG measurements suggest that individuals in HDtDCS-group showed better neuromuscular control when performing hand movements after the intervention compared with Sham-group.

## Discussion

To our knowledge, this pilot study is the first randomized controlled trial to investigate the synergetic effect of personalized HD-tDCS on chronic stroke survivors. This study demonstrated the optimization of the personalized HD-tDCS on the individual motor hotspot of chronic stroke survivors navigated by tb-fMRI. The clinical and neuroimagings measures showed consistent findings that revealed the additive stimulation effect, which might be associated with the prior motor improvement in HDtDCS-group.

Using structural and functional MRI with FEM to optimize the HD-tDCS has been applied in healthy and stroke subjects [27, 28, 30]. Our results also demonstrated the feasibility of optimizing HD-tDCS for post-stroke rehabilitation. The optimization performance which was evaluated by the ERNI value was in line with previous optimization study [28]. Different from using resting-state fMRI connectivity as the optimization target map, we optimized the montages navigated by hand-task-based brain activation, as we can precisely target the post-stroke individual motor hotspots while performing grasping hand tasks. The task was exactly repeated during the EMG-RH training. With this design, we were able to link the HD-tDCS and EMG-RH and investigated how stimulation prime additional effects on the cortical excitability besides that induced by EMG-RH.

Our research results align with our hypothesis that motor function may be enhanced through personalized HD-tDCS before EMG-RH training. The improvement after the robotic hand was illustrated in previous studies, including the gains in clinical scores, finger dexterity, and muscle coordination [31, 54]. The EMG-RH training was triggered by voluntary muscle contraction, and the assistance of the robotic hand gave feedback on the intention of moving their hands, the whole process was an active closed loop. Thus, it is not surprising that Sham-group also showed increased lateralization of ipsilesional cortical activation and cortico-muscular connection after training. The FMAUE and MASf scores indicated HDtDCS-group gained greater improvement in UE function and finger spasticity. These preliminary findings imply that HD-tDCS may have add-on effects in facilitating motor recovery in addition to EMG-RH training alone.

To investigate the stimulation effects on the central-peripheral alterations, our study employed multiple neuroimaging techniques, including fMRI, EEG, and EMG. Through these multimodal measurements, we have pinpointed three potential neural modulation effects that are linked to UE motor recovery: (1) reorganization of interhemispheric sensorimotor cortex activation, (2) facilitation of cortico-peripheral connections between the primary motor cortex and muscle, (3) improved peripheral muscle coordination. The tb-fMRI showed that HDtDCS-group had greater increases in $\:LI$ value during ME tasks after intervention than Sham-group and the significant improvement was observed after 6 months (Fig. 4c). Previous research showed that stroke survivors exhibited lower BOLD activities in the ipsilesional hemisphere and increased activities in the contralesional hemisphere when performing tasks with paretic hands, resulting in a lower $\:LI$ compared to healthy individuals [57]. The interhemispheric rebalance in the sensorimotor cortex was thought to reflect the motor recovery after stroke [42, 58, 59]. The current results showed a similar pattern of brain activation changes across hemispheres (Fig. 4b, c). Interestingly, we found the HDtDCS-group presented continued increases in *LI* at *6 m Follow-up*, and the sustained ipsilesional activated regions were stimulated in the group-level EFs from Fig. 4a. In contrast, the *LI* value in the Sham-group dropped back towards the *Post* session by the *6 m Follow-up.* This result suggests that synergetic effects induced by the HD-tDCS might prompt long-lasting positive effects on neuroplasticity. To verify this idea, we further conducted *Specificity* calculation to explore the relationship between hand-task brain activations and stimulated regions. As shown in Fig. 4e, the specificity value increased after intervention and continued to increase at the *6 m Follow-up* in HDtDCS-group, but not in the Sham-group, which suggests there was more overlap between the EF and activation map after training in HDtDCS-group. These results potentially explain the greater increase in $\:LI$ values for the HDtDCS-group, as the activation map in HDtDCS-group seemed to be concentrated in the area facilitated by HD-tDCS, and this facilitation was sustained at the follow-up evaluation. The higher $\:LI$ values after training suggested that subjects might have relied more on the ipsilesional sensorimotor cortical activation during paretic hand movements, which could be linked to better motor function improvement [60, 61]. The continuous increase of *LI* value was also consistent with sustained improvement of FMAUE scores in HDtDCS-group (Fig. 5a). The possible explanation for brain facilitation and motor recovery for HDtDCS-group is that the personalized stimulation was delivered specifically to the ipsilesional hand-task sensorimotor hotspots. The online (modifying local cortical excitability [62]) and offline effects (LTP-like effects) of HD-tDCS could have modulated interneuron activity and postsynaptic receptor efficacy [63], likely contributing to the facilitation of the ipsilesional cortical activation and additional motor recovery that was observed at Follow-up assessment. We observed a large effect size (Cohen’s d = 1.11) in the tb-fMRI result of HDtDCS-group, but it was from a small sample size, and the P value in fMRI results was close to the significance threshold. We should consider a larger study in the future to evaluate the effectiveness.

Our results also showed increases in CMC after training, which potentially indicated the facilitated motor control and motor recovery. Similar to the tb-fMRI results, $\:Beta{CMC}_{EDopen}$ continued increase in HDtDCS-group at *6 m Follow-up* (Fig. 6d and f), while that of Sham-group slightly dropped. Although the CMC data did not show significant between-group interaction after training, the greater increase in CMC values of HDtDCS-group suggests that the HD-tDCS might have enhanced the functional connection between the cortex and muscles. We also noticed that the $\:Beta{CMC}_{FDgrasp}$ in HDtDCS-group showed a slight decrease at *6 m Follow-up*, although it was still significantly higher than the *Pre* assessment. These results might be related to a difficulty-dependent pattern of the CMC variable, where tasks with lower difficulty tend to produce smaller CMC values [64, 65]. We also observed a continued decrease in the co-contraction index between FD and ED (Fig. 6e) in our EMG assessment at *6 m Follow-up*. This suggested that subjects were able to perform hand tasks with more coordinated muscle contraction and better motor control [54], making the tasks less difficult for them. These EMG results could partially explain the slight decrease observed in $\:Beta{CMC}_{FDgrasp}$ at *6 m Follow-up*. Despite the slight drop at *6 m Follow-up*, the changes in CMC value were consistent with FMAUE scores, which also showed increases at *Post* and *6 m Follow-up* when compared to baseline, while no significant difference was observed between *Post* and *6 m Follow-up* assessments.

Another key finding was the significant reduction in $\:{CI}_{open}\:$ and MASf scores in HDtDCS-group, compared to Sham-group. The results suggested that personalized HD-tDCS may be beneficial for post-stroke spasticity management. The reduced spasticity after the intervention could be associated with improved reciprocal inhibition and relief of the stretch reflex. Reciprocal inhibition is a neural phenomenon in the human body where, when the agonist muscle contracts, impulses from Ia inhibitory interneurons in the spinal cord inhibit the tension of the antagonist muscle, resulting in its relaxation [66]. Task-oriented robotic hand training has been reported to promote inhibitory control of flexors and improve reciprocal inhibition, thus helping stroke survivors relearn control of intended movements and facilitating the function of antispastic motor neurons [67, 68]. Moreover, the greater reduction in spasticity for HDtDCS-group could be attributed to the personalized HD-tDCS. Previous studies have found that tDCS could have adjunctive effects in reducing post-stroke spasticity [69–71]. The hyperexcitability of the stretch reflex induced by the disinhibition of the disrupted efferent circuits (dorsal cortico-reticulospinal tract, dorsal cortico-RST) is the main neuronal factor of post-stroke spasticity [72]. When lesions occur in the motor cortex and cortical-RST, inhibition of the stretch reflex circuitry diminishes, ultimately resulting in hyperexcitability or spontaneous firing of the stretch reflex circuitry together with the spinal motor neurons [73]. The potential mechanism underneath HD-tDCS treating spasticity is that the LTP-/LTD- like mechanism could upregulate the inhibition effect of cortical-RST on stretch reflex, which contributed to lower muscle spasticity. However, conventional tDCS mostly targets the M1, but the cortico-RST originates not only from M1 but also the SMA and PMC. One advantage of the personalized HD-tDCS is that it could precisely stimulate these motor hotspot, as demonstrated in Fig. 3b.

### Clinical implication

While personalized HD-tDCS offers significant benefits, the additional time, cost, and risks should be considered. The 20-session combined intervention required approximately 1640 min (~ 27.3 h), which included MRI scanning (around 40 min) and 20 sessions of intervention (20-minute HD-tDCS and 60-minute robotic hand in each session, around 1600 min in total). This resulted in an extra cost of around 1500USD, including one-session MRI scanning (500USD) and associated manpower in public clinical settings (50USD/session and 1000USD in total). Moreover, HD-tDCS is generally safe when operated with certified devices within the limited current intensity [74, 75], while if subjects cannot comply with MRI protocol, such as claustrophobia, they should not be able to participate in this MRI-involved HD-tDCS intervention protocol.

### Limitations and future work

Although our data showed some promising results, several limitations should be acknowledged. Firstly, the small sample size in this pilot randomized controlled trial is a limitation. The small sample size may have limited the generalizability of these findings, even though the trends observed in the clinical scores, fMRI, EEG-EMG, and EMG assessments were similar. Future research will be needed to validate these results with a larger sample size to strengthen the confidence in the findings. Additionally, the current study focused on chronic stroke survivors. We also recommend conducting future studies that include sub-acute stroke survivors to evaluate the intervention effects during the early stages of rehabilitation. Secondly, the lack of a control group adopting conventional tDCS with EMG-RH prevented us from drawing definitive conclusions about the superior neuromodulatory effect of HD-tDCS over conventional tDCS. We recommend having conventional tDCS in the future study. In this study, we aimed to investigate the additional effect of delivering 20 sessions of personalized HD-tDCS before EMG-RH compared to EMG-RH only, and we provided some preliminary results to demonstrate the feasibility and potential effectiveness of personalized HD-tDCS on motor function improvement.

## Conclusion

By precisely targeting the individual motor hotspot identified through tb-fMRI, our study demonstrates that brain activation in the specifically stimulated regions can be further enhanced by HD-tDCS. These findings suggest that personalized HD-tDCS has the therapeutic potential for post-stroke UE motor rehabilitation. However, it is important to note that further validation through randomized controlled trials with a large sample size is necessary.

## Electronic supplementary material

Below is the link to the electronic supplementary material.

Supplementary Material 1

## Data Availability

All necessary data supporting these results are available in the figures and text from manuscript and supplementary file. If readers have any further questions regarding the data and results, they can contact the corresponding author.
