# Supplementary material for: Effects of high-definition tDCS targeting individual motor hotspot with EMG-driven robotic hand training on upper extremity motor function: a pilot randomized controlled trial
Source: J Neuroeng Rehabil. 2024 Sep 20;21:169. doi: 10.1186/s12984-024-01468-w (PMC11414071; doi:10.1186/s12984-024-01468-w)
Supplement: Supplementary file 1 — Supplementary Material 1 [file 12984_2024_1468_MOESM1_ESM.docx]

**Supplementary Materials**

**Effects of High-definition tDCS Targeting Individual Motor Hotspot with EMG-driven Robotic Hand Training on Upper Extremity Motor Function: A Pilot Randomized Controlled Trial**

**Chengpeng Hu^1*^, Chun Hang Eden Ti^1*^, Kai Yuan^1^, Cheng Chen^1^, Ahsan Khan^1^, Xiangqian Shi^1^, Winnie Chiu-wing Chu^2^, Raymond Kai-yu Tong^1#^**

1. Department of Biomedical Engineering, The Chinese University of Hong Kong, Hong Kong SAR, China

2. Department of Imaging and Interventional Radiology, The Chinese University of Hong Kong, Hong Kong SAR, China

*: These two authors contributed equally to this study.

Corresponding author:

Raymond Kai-yu Tong, Department of Biomedical Engineering, The Chinese University of Hong Kong, Hong Kong SAR, China. Email: [kytong@cuhk.edu.hk](mailto:kytong@cuhk.edu.hk)

**1. Supplementary methods**

**1.1 MRI data acquisition and preprocessing**

MRI scans were acquired using a 3T Siemens Prisma MRI scanner (Siemens Healthcare, Erlangen, Germany) with an 8-channel head coil, including T1-weighted anatomical images (TR/TE = 1900/2.93ms, flip angle = 9°, 176 slices, voxel size = $0.9\times0.9\times1.0 {mm}^{3}$) using a T1-MPRAGE, and BOLD fMRI images ( TR/TE = 1200/ 30 ms, flip angle = 68°, 48 slices, voxel size = $3.0\times3.0\times3.0 {mm}^{3}$) using an EPI-FID sequence. The sequences for task-based fMRI were displayed using EPrime 3.0 (Psychology Software Tools, PA USA). Motor execution (ME) tasks were designed based on the EMG-RH training. During the ME task, two tennis balls were placed in the subject’s left hand and right hand respectively in advance. In case of the ball fell out of the affected hand, adhesive tape was used to fix the tennis ball in the affected hand. Subjects were asked to grasp the corresponding hand when a mark of “left hand” or “right hand” appeared on the screen and were asked to maintain 6 seconds until a “rest” mark appeared. An event-related design was adopted with a randomized inter-trial interval ranging from 12 to 20 seconds. A total of 20 tasks were randomized adopted, leading to around 6.8 minutes tb-fMRI were performed for one scanning session. Three tb-fMRI scannings were performed for each subject at *Pre*, *Post*, and *6m Follow-up.*

The tb-fMRI imaging were preprocessed using DPARSF toolbox. To make the lesion side consistent among the participants, the imaging of subjects with the lesion on the left hemisphere was flipped along the midsagittal plane so that the lesions of all participants were normalized in the right hemisphere. Firstly, the first 10 volumes were discarded to ensure the remaining volumes of the fMRI data were acquired at magnetization steady state. The remaining volumes were corrected with slice timing and realigned for head motion correction. Nuisance variables were then regressed out, including white matter, cerebrospinal fluid (CSF), global mean signal, and Friston 24 head motion parameters ^1^. To further control for head motion, the scrubbing process was performed for the volumes with framewise displacement (FD) value exceeding 0.7 ^2^. If over 25% of all the volumes exceed the FD threshold, the data for this subject would be discarded. Then the functional dataset was aligned to the anatomical dataset. Subsequently, the functional images were spatially normalized to the Montreal Neurological Institute (MNI) template, resliced to 2 × 2 × 2 mm^3^ voxels, and smoothed with a Gaussian kernel with a full-width at half-maximum (FWHM) of 6 mm.

After preprocessing, the data were fitted into a general linear model for subject-level analysis. Each of the events was convolved using a canonical hemodynamic response function (HRF) and used as a regressor. As a result, a *t-map* was generated for each subject at *Pre, Post, 6m Follow-up* sessions. One subject in Sham-group was excluded from the analysis because of excessive motion artifacts. In addition, the MRI data acquired before intervention were also used to generate personalized HD-tDCS montages. Specifically, the t-maps acquired at Pre were recognized as the individual motor hotspot allocation, and the structural MRI acquired at Pre was used to determine individual lesion profiles and brain structure information.

**1.2 Personalized stimulation montage for HD-tDCS**

The procedures for optimizing individualized stimulation montages were performed using SIMNIBS3.4.0 ^3^. The first step is to generate individualized finite element model. A FEM was generated from individual structure T1 and T2 images. Each FEM consists of six compartments, namely the scalp, skull, cerebrospinal fluid (CSF), grey matter (GM), white matter (WM), and stroke lesions (Figure 1.1a). Non-brain tissues were assigned with standard conductivity values, whereas conductivity values of GM and WM were estimated from individual DTI images using a volume-normalized approach ^4, 5^. Optimization of montages was then performed on individual FEM models following the procedures described in previous studies ^6, 7^. Briefly, an arbitrary reference electrode was defined at CZ as the cathode. EF simulations were then performed over 39 electrode positions available on the StarStim cap (Neoprene Headcap, Neuroelectrics, Barcelona, Spain). Each simulation consisted of a bipolar electrode configuration with the anode at a specific location and the reference applied at 1mA, resulting in a leadfield matrix consisting of 39 EF simulation results over the FEM model. The electrodes were modeled as Ag/AgCl electrodes with 2 cm diameter and 12 mm thickness based on the corresponding electrodes (NG Pistim, Neuroelectrics, Barcelona, Spain). With the leadfield matrix, EFs for any defined montages were expressed as a linear superposition of the leadfield and their corresponding intensity (Figure 1.1b). The optimization was simplified to find the electrode and intensity that


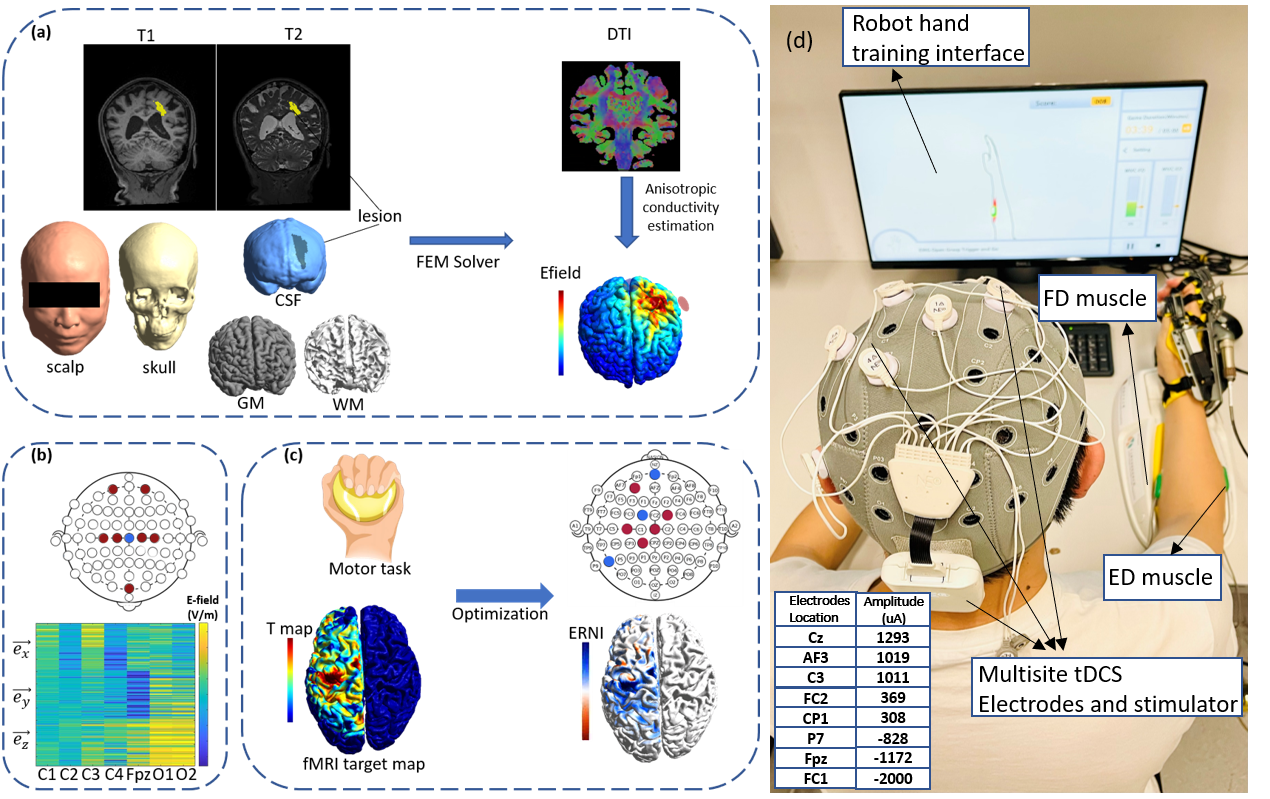
Figure 1.1 demonstrates the optimization procedures and training protocols of the current study. (a) Generation of individualized finite element model. Simulation of the electric field was performed by solving the Laplace Equation using FEM solver after placing modeled electrodes at the desired locations. (b) Generation of leadfield matrix. Leadfield was generated by performing simulations with bipolar configurations, with anodes(red) placed at a defined set of locations(39 in total) and Cz(blue) as the cathode. The figure showed seven channels for illustration purposes. The column of the matrix represents the electric field in $\vec{e_{x}}, \vec{e_{y}},\vec{e_{z}}$ direction over the volume conductor models. (c) Optimization of tDCS montages. Stimulation targets were defined using individual fMRI activations during paretic motor tasks. Optimization was based on a distributed constrained maximum intensity method, which minimizes the Error Relative To No Intervention(ERNI). (d) A stimulation montage containing the amplitude and location of each electrode for one subject was generated. One subject sat in front of the screen and performed robotic hand training.

generated the EF patterns that reach the defined target (Figure 1.1c). Specifically, the individual fMRI statistical t-map from the ME tasks was used to localize the active regions during paretic hand motor tasks. The map was interpolated to the cortical surface to generate a t-value on each node of the FEM. Nodes with t > 2.3 were defined as the target for optimization. Following the procedures described in reference ^6^, the optimization minimizes the sum of Error Relative To No Intervention(ERNI) described in formula (1):

$$ERNI\left( x \right)=\frac{\sum_{x} \left( \left( Y_{\omega}\left( x \right)-E_{\omega}\left( x,M \right) \right)^{2}-Y_{\omega}\left( x \right)^{2} \right)}{\frac{1}{N_{x}}\sum_{x} W_{x}^{2}} \ldots\ldots\ldots(1)$$

Here, *x* represents the node of the FEM. $Y_{\omega}(x)$ is the weighted desirable EF. The desirable EF is zero for non-target nodes and 0.3 V/m normal to the cortical surface for target nodes to promote excitatory responses. The normal EF component was considered in this study because EFs in this direction were shown to induce a larger increase in cortico-excitability. To assure higher importance in the more activated region, the target EFs were weighted by the t-value of each node, denoted by $W_{x}$. The $N_{x}$ represents the total number of nodes in the FEM. Under given montage *M*, this equation quantifies the deviation of the weighted target $Y_{\omega}(x)$, and the same-weighted normal EF component $E_{\omega}(x,M)$ compared with no stimulation at all. A negative value represents EFs at the node approaching the target EFs, indicating a better fit. This approach weighs heavily on highly activated regions and penalizes non-target stimulation ^6^. Montage M was set with the following constraints: 1) The total number of electrodes is less than or equal to 8. 2) The total injected current inside the brain does not exceed 4mA. 3) The injected current of each electrode does not exceed 2mA. 4) The sum of the total current equals zero. The results of optimization were quantified by the total ERNI and the weighted cross-correlation (WCC) between the target EF and simulated EF, using the definition in a previous study ^6^.

**1.3 HD-tDCS combined EMG-RH training**

Each subjects underwent 20 sessions of HD-tDCS combined EMG-RH training, with an average of 1-3 sessions delivered each week. For each training session, HD-tDCS was conducted before EMG-RH training, where subjects received 20-minute personalized stimulation with the optimized stimulation montages, including a 1-minute ramp-up and ramp-down period. For the Sham-group, only ramp-up and ramp-down stimulation was applied.

After HD-tDCS, EMG-RH training was conducted for both groups using EMG-driven linear-motor robotic-hand system (Hand of Hope, Rehab-Robotics Co. Ltd, Hong Kong SAR, China). Each session contained three blocks of training with 15 minutes training in each block. Between two blocks, subjects could take 5-minute rest. Subjects were told to sit comfortably in front of a computer and wore the robot hand on their paretic hands with the forearm resting on a table and elbow slightly flexed at about 130°. After skin preparation, EMG electrodes were placed over the flexor digitorum (FD) and extensor digitorum (ED), and the reference electrode was placed over the olecranon process of the paretic arm (Figure 1.1d). Before each training session, baseline and maximum voluntary contraction (MVC) signals from the FD and ED muscles were collected. Each MVC was maintained for five seconds for two attempts. During EMG-RH, linear actuators on the robotic hand would provide active powered assistance for repetitive hand grasping and opening tasks. The robotic-hands provided 55-degree and 65-degree range of motion for metacarpophalangeal and proximal interphalangeal joints respectively to enable functional finger movements. A simulated hand was grasping or opening on the screen and there was also a notification (“grasp your hand” or “open your hand”) to remind subjects of contracting relative muscles. The EMG signals collected from voluntary contraction of the FD and ED muscles were used to trigger the grasping and opening of the robotic hand, respectively. The real-time EMG signals were collected (sampling frequency of 1000Hz), amplified (gain of 1000), processed and enveloped as EMG-level following standardized procedures (50Hz notch, 10-400Hz band-pass, rectification, and 5 Hz low-pass filtering). Each power-assisted movement would take 5 seconds to complete. Movements were triggered when the 100ms averaged EMG-level exceeded pre-set threshold (10% of the MVC measured before each session). Based on our previous studies, the threshold to trigger the grasping/opening of the robotic hand was set at 10% of MVC ^8^, at which our chronic stroke participants could comfortably and consistently trigger the activation of the robotic hand with their residual EMG during voluntary contraction. The threshold can be adjusted based on the participant’s performance and feedback to meet the best intervention effect by the experienced experimenter. Our group member who is a licensed physical therapist conducted the robotic hand intervention for subjects through the entire training process. This therapist educated the subjects on how to correctly control the robotic hand, ensuring they understand how to generate voluntary muscle contractions above the threshold to trigger the robotic movements. During the assistive training, subjects were instructed to keep contracting muscles until the robot handstoppeds, and then perform the next movement following the instruction on the screen. The details of the robot hand training were demonstrated in our previous study ^8^.

**1.4 Task-based fMRI parameter calculation**

**Lateralization Index calculation**

A voxel-level threshold of *p* < 0.001 was applied and corrected by Gaussian random field theory at the threshold of *p* < 0.05 at the cluster level. The voxels located in the sensorimotor areas (motor, premotor, and somatosensory regions) were masked for the $LI$ calculation ^9^. The $LI$ value was computed using formula (2).

$$LI= \frac{N_{ipsi} - N_{contra}}{N_{ipsi} + N_{contra}} \ldots\ldots\ldots(2)$$

where $N_{ipsi}$ stands for the number of activated voxels in the ipsilesional hemisphere. $N_{contra}$stands for the number of activated voxels in the contralesional hemisphere. Therefore, the range of $LI$ value was between -1 and 1. A LI value of 1 represents the activation lies purely in the ipsilesional hemisphere and -1 represents the activation purely relies on the contralesional hemisphere.

**Specificity calculation**

On the FEM models of each individual in the HDtDCS-group group, we first defined the masks of the stimulated region ($E_{i})$ by thresholding EFs by values larger than the 50^th^ percentile of the overall EF distribution on the GM surface. The GRF-corrected activation maps at *Pre, Post, and 6m Follow-*up sessions were projected on the same FEMs. Finite elements with t > 2.3 were included as the corresponding activation masks ($A_{ij}$). With the defined mask, we used the F score to quantify the Specificity of the HD-tDCS effect as formula (3):

$${Specificity}_{ij}=\frac{2\left( N_{\left( E_{i} | A_{ij} \right)}\times N_{\left( A_{ij} | E_{i} \right)} \right)}{N_{\left( E_{i} | A_{ij} \right)}+N_{\left( A_{ij} | E_{i} \right)}} \ldots\ldots\ldots(3)$$

Where i and j correspond to individual and session respectively. $N_{\left( x | y \right)}$ represents the ratio of the sum of finite elements over regions shared by x and y to the sum of finite elements over region y. Specifically, $N_{\left( E_{i} | A_{ij} \right)}$ represents the proportion of activated regions that are being stimulated by tDCS, and $N_{\left( A_{ij} | E_{i} \right)}$ represents the proportion of activation given the stimulated regions. Specificity ranged from 0 to 1, with value 1 representing a perfect overlap between the electric field and activation masks, and value 0 representing no overlapping of the masks.

**1.5 EEG-EMG measurement and Cortico-muscular coherence (CMC)**

To evaluate the connection between central neural system and peripheral muscles during motor task, EEG-EMG assessments were conducted at Pre, Post, and 6m Follow-up assessments for each subject. During the data acquisition, the 128-channel Neuroscan amplifier (SynAmps2, Neuroscan Inc, Herndon, USA) was used to collect EEG and EMG signals. and a 128-channel Quik-Cap EEG cap was used for EEG acquisition. Two pairs of bipolar EMG electrodes were carefully placed over the affected FD and ED muscles, and the collected data were used for CMC calculation. The sampling frequency was 1000 Hz. A separate 2-channel g.USBamp (g.tec Medical Engineering GmbH, Austria) was utilized to collect EMG signal from FD/ED muscle for visual feedback, these two pairs of EMG electrodes were attached beside the above-mentioned Neuroscan EMG electrodes to provide real-time feedback for participants to control force outputs. Two computers were used, one for recording the EEG/EMG signals using Neuroscan Curry 7 software ((Neuroscan Inc, Herndon, USA) and the other for Matlab ((Mathworks Inc., Natick, MA, USA) online feedback interface. The two computers were synchronized using parallel port communication, and TTL triggers were sent to the Neuroscan amplifier via Matlab to mark the beginning and end of each trial.

The CMC measurements consisted of two tasks: isometric contraction of FD and ED, respectively. Participants were instructed to sit comfortably, with eyes open and focusing on the center of the screen in front of them while the forearms were placed on the table. To ensure participants maintained a steady 30% MVC of grasping and opening their hands, an online EMG feedback system was used, and subjects were instructed to control the blue bar in the allowed error region. During the FD isometric contraction task, the experienced research staff held against the direction of the subjects’ finger flexion and stabilized their hand and wrist to prevent joint movements. During the ED isometric contraction task, the research staff held against the direction of the subjects’ finger extension and stabilized their hand and wrist as well. The details of the EMG feedback system were previously described in Bao’s study ^10^. To ensure consistency among participants, the FD task was performed first. Each task lasted around 5 minutes, including 3 contraction trials and two 1-minute intermediate breaks. Each trial lasted 40 seconds. After the FD task, participants rested for 5 minutes before the ED task. The EEG experiment workflow and feedback interface are depicted in Figure 1.2.

Before measuring the CMC, the EMG signal of MVC was collected for FD and ED, respectively. During the MVC measurement, the subjects were asked to fully grasp and open their affected hands for 8 seconds while an experienced research staff held against the subjects’ hand to ensure subjects could perform MVC smoothly. The maximum EMG magnitude was determined by averaging the rectified EMG signals during the MVC. The MVC of FD and ED was utilized to guide subjects perform stable muscle contraction in the tasks, in which 30% of MVC was set according to our previous study ^10^. After 5-minute rest, subjects were instructed to perform 40-second grasping hand for 3 times and 40-second opening hand for 3 times with 5-minute rest between these two tasks. The workflow of measurement was demonstrated in Figure 1.2a. An online EMG feedback interface was shown in front of subjects to ensure the force stability maintained at around 30% of MVC (Figure 1.2b). Subjects were instructed to exert muscle contraction and keep the blue bar in the allowed error region between two bold black lines. The blue bar swayed with a lower pivot endpoint according to the EMG amplitude. The angle of the bar was defined in formula (4), and the bold black lines represented the 10% allowed error range.

$Err\%= \frac{{EMG}_{avg}-0.3 \times{EMG}_{MVC}}{0.3 \times{EMG}_{MVC}} \times100\%$ … … … (4)


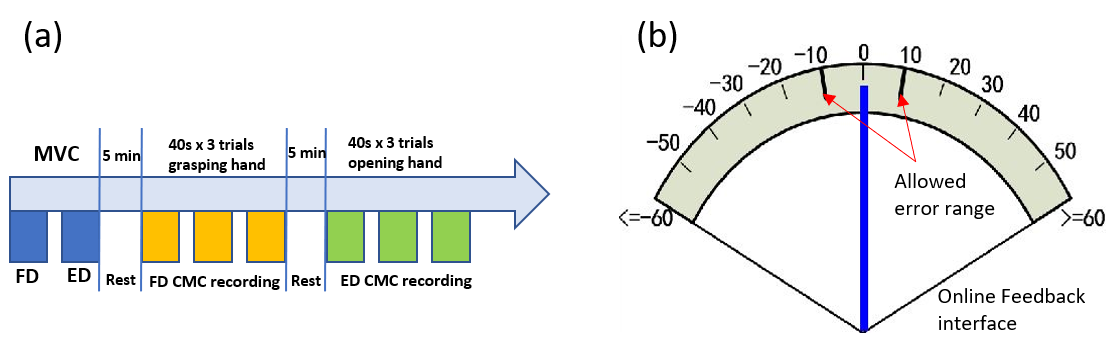


Figure 1.2 The work flow of CMC measurement (a), and the online EMG feedback interface (b).


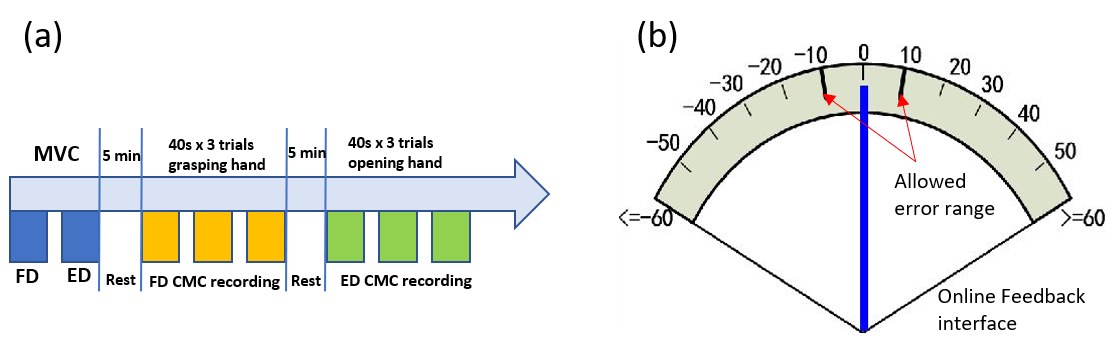


Figure 1.2 The work flow of CMC measurement (a), and the online EMG feedback interface (b).

The EEG and EMG signals were aligned for the isometric contraction timelines. EMG signals were processed using a bandpass filter (2-400 Hz), and a 50 Hz notch filter. The channels near the EEG-cap border and ears (e.g. T9, FT9) were excluded to avoid poor gel contact, leaving 85 EEG channels were selected for further analysis. The EEG signals were downsampled to 500 Hz, baseline corrected, 1-45 Hz bandpass filtered to remove signal drifts, and finally common average referenced. The ipsilesional hemisphere was standardized to the right hemisphere to ensure consistency among participants. For subjects with a lesion on the left hemisphere, the left channels were flipped across the midline to the corresponding right channels (e.g. original C3 was the new C4, while the Cz channel was not shifted), and the paretic limbs were normalized to the left side accordingly. No channel shifting was applied for participants with a lesion on the right hemisphere. Independent component analysis (ICA) was performed using the three-model adaptive mixture independent component analysis (AMICA) algorithm ^11^, followed by a multiple artifact rejection algorithm (MARA) to obtain cleaned EEG signals ^12^. For each task (3 trials), a total of 120 seconds of signals were divided into non-overlapping segments with 512 sample points per segment. The first and last segments in each trial were abandoned due to signal instability. Channels and segments with poor quality were removed by manual summary-based artifact rejection using the Fieldtrip toolbox, leaving about 100-111 segments in each task. Finally, a spline surface Laplace filter was applied to reduce volume conduction and improve EEG focality ^13^. EMG signals were downsampled to 500 Hz, rectified, and stratified with a 200 ms boxcar filter to minimize signal fluctuation ^14^.

The magnitude-squared coherence spectrum was calculated based on the power spectral density estimation with formula (5),

$$C_{XY}\left( F \right)=\frac{||P_{XY}{(f)}^{2}}{P_{XX}(f)P_{YY}(f)} \ldots\ldots\ldots(5)$$

where $P_{XX}(f)$ and $P_{YY}(f)$ were the auto-power spectral density (PSD) of EEG and EMG signals (represented as X and Y) throughout segments for a given frequency f, and $P_{XY}(f)$ is the cross-power spectral density between them.

The area of stroke survivors' CMC-related motor function might shift away from the ipsilesional M1 area as reported in previous studies ^15^. To mitigate this effect, a cluster of five channels (C4, FCC4H, FCC6H, CCP4H, and CCP6H) located at the primary motor cortex, was selected as the target region. The frequencies of interest were defined as the Alpha band (8 -13 Hz), Beta band (13 - 30 Hz), and low Gamma band (30 – 45 Hz). The CMC value was defined as the “Peak” coherence, namely the largest coherence in the given frequency band. The CMC topographies generated at the peak-CMC-relative frequency from selected channels were averaged and demonstrated in different frequency bands at three time sessions. To better understand the motor control improvement when subjects perform grasping and opening hand tasks, we calculated ${CMC}_{FDgrasp}$ and ${CMC}_{EDopen}$ in three frequency band.

**1.6 Signal processing for EMG data**

Before the assessment, surface EMG electrodes were placed on affected FD and ED following standard skin preparation. The reference electrode was attached to the skin surface above the olecranon process. MVCs from FD and ED were collected to normalize the EMG signals. Followed by the MVC recording, participants were instructed to perform unassisted, repetitive, full hand grasping (4s), hand opening (4s), and rest (4s) for ten cycles with a comfortable muscle contraction while following visual cues displayed on a computer screen.

All the EMG signals were sampled with 1000 Hz, and stored for off-line processing. The standard processing methods were as follows: firstly, the linear envelope of the recorded EMG signals was calculated by: (i) full-wave rectification, (ii) low-pass filtering (fourth-order, zero-lag Butterworth filter with 10 Hz cut-off frequency), (iii) subtraction of the average EMG activity during the resting state, and (iv) normalized to the maximum value of EMG activation in MVCs. Then, the five cycles in the middle were extracted for data analysis after visual inspection.

Muscle coordination was determined using CI as computed with formula (6)

$${CI}_{k}= \frac{1}{T}\int_{0}^{T} A_{k}\left( t \right)dt \ldots\ldots\ldots(6)$$

where $A_{k}$ was the overlapping activity of EMG linear envelopes for the FD/ED muscle pair during the movement *k* (i.e. hand grasping and opening), and *T* was the length of the signal. The CI of FD/ED muscle varied from 0 (no overlapping of muscle contractions) to 1 (complete overlapping of two maximal muscle contractions with both EMG activation levels kept at 1 during relative movement). A higher CI value indicates an enlarged co-contraction phase of two muscles during the movement, which leads to less energy-efficient joint movement, whereas a lower CI suggests a separation in the co-contracting phase of two muscles which leads to improved muscle coordination pattern during a movement ^8, 16^.

**2. Supplementary Results**

The details of the demographic information of subjects are demonstrated in Table 2.1, including the lesion volume and the lesion location. Table 2.2 depicites the details on the location and intensity of each electrode in individual stimulation montage. Table 2.3 demonstrates the comparison of clinical scores in two groups. Table 2.4 depicites the detail CMC variables comparison. Table 2.5 depicites EMG variables comparison of two groups. Table 2.6 demonstrates the comparison of clinical demographic information of HDtDCS-group and Sham-group groups, because one subject from HDtDCS-group group dropped out after three-session of training, we conducted another baseline comparison, and there were no significant differences in clinical information between HDtDCS-group and Sham-group groups.

**Table 2.1 Information of location and intensity for personalized stimulation montages**

| **Channel list** | **Ch 1** | **Ch 2** | **Ch 3** | **Ch 4** | **Ch 5** | **Ch 6** | **Ch 7** | **Ch 8** |  |
| --- | --- | --- | --- | --- | --- | --- | --- | --- | --- |
|  |  |  |  |  |  |  |  |  |  |
| **HDtDCS-group Subj 01** | CP1 | Cz | AF7 | T7 | P7 | F3 | Fz | / |  |
|  | 2.000 | 1.255 | 0.745 | -0.820 | -1.030 | -1.030 | -1.120 | / |  |
| **HDtDCS-group Subj 02** | PO3 | C1 | T7 | Fp1 | O1 | F7 | PO7 | FC1 |  |
|  | 1.443 | 1.414 | 0.821 | 0.321 | -0.363 | -0.485 | -1.531 | -1.620 |  |
| **HDtDCS-group Subj 03** | C3 | C1 | FC2 | Fpz | CP5 | Pz | FC1 | / |  |
|  | 1.661 | 1.495 | 0.690 | -0.305 | -0.790 | -0.918 | -1.833 | / |  |
| **HDtDCS-group Subj 04** | Cz | AF3 | C3 | FC2 | CP1 | P7 | Fpz | FC1 |  |
|  | 1.293 | 1.019 | 1.011 | 0.369 | 0.308 | -0.828 | -1.172 | -2.000 |  |
| **HDtDCS-group Subj 05** | C3 | FC5 | PO3 | C2 | F7 | CP5 | FC1 | Pz |  |
|  | 1.460 | 0.966 | 0.803 | 0.771 | -0.646 | -0.839 | -1.141 | -1.374 |  |
| **HDtDCS-group Subj 06** | C2 | C4 | T8 | F8 | PO8 | CP6 | FC2 | / |  |
|  | 1.589 | 1.442 | 0.969 | -0.310 | -0.536 | -1.154 | -2.000 | / |  |
| **HDtDCS-group Subj 07** | C2 | PO4 | F8 | AF4 | P8 | F7 | FC2 | PO8 |  |
|  | 1.361 | 1.069 | 0.772 | 0.222 | -0.245 | -0.660 | -1.063 | -1.456 |  |
| **HDtDCS-group Subj 08** | FC6 | P4 | Cz | FC1 | F4 | P8 | FC2 | / |  |
|  | 1.573 | 1.016 | 0.971 | 0.334 | -0.624 | -1.517 | -1.753 | / |  |
| **Sham-group subj 01** | C3 | CP1 | C2 | T7 | Pz | PO3 | F7 | FC1 |  |
|  | 1.991 | 0.739 | 0.698 | 0.572 | -0.516 | -0.688 | -0.796 | -2.000 |  |
| **Sham-group subj 02** | C1 | FC5 | P3 | FC2 | P7 | Oz | C3 | FC1 |  |
|  | 1.561 | 1.245 | 0.831 | 0.363 | -0.393 | -0.565 | -1.290 | -1.752 |  |
| **Sham-group subj 03** | FC5 | C1 | FC2 | CP1 | F3 | T7 | FC1 | / |  |
|  | 1.969 | 1.625 | 0.406 | -0.541 | -0.747 | -1.247 | -1.465 | / |  |
| **Sham-group subj 04** | C2 | FC1 | T8 | P4 | FC5 | F4 | Fz | CP2 |  |
|  | 1.820 | 1.812 | 0.368 | -0.325 | -0.365 | -0.757 | -0.876 | -1.677 |  |
| **Sham-group subj 05** | C3 | CP1 | T7 | Cz | PO7 | CP5 | FC1 | / |  |
|  | 1.594 | 1.153 | 0.764 | 0.370 | -0.526 | -1.474 | -1.881 | / |  |
| **Sham-group subj 06** | T8 | C2 | PO4 | FC1 | F7 | FC2 | P8 | / |  |
|  | 1.470 | 1.202 | 0.686 | 0.642 | -0.619 | -1.349 | -2.032 | / |  |
| **Sham-group subj 07** | C1 | C3 | T7 | FC2 | FC5 | P7 | FC1 | / |  |
|  | 1.885 | 0.846 | 0.828 | 0.441 | -0.843 | -1.157 | -2.000 | / |  |
| **Sham-group subj 08** | FC1 | F4 | CP2 | Fp2 | C3 | F3 | AF4 | FC2 |  |
|  | 1.519 | 0.816 | 0.689 | 0.599 | -0.430 | -0.476 | -0.718 | -2.000 |  |
| **Sham-group subj 09** | FC2 | F8 | P8 | F4 | C4 | Fz | / | / |  |
|  | 1.847 | 0.968 | 0.696 | -0.818 | -1.210 | -1.483 | / | / |  |
| **Sham-group subj 10** | C3 | CP1 | C1 | T7 | FC5 | CP5 | P3 | FC1 |  |
|  | 1.965 | 0.986 | 0.735 | 0.313 | -0.268 | -0.882 | -1.284 | -1.565 |  |

Ch: Channel; Subj: Subject; The location of electrode is using international 10-20 EEG system. Unit of the stimulation intensity is mA. The electrode order was arranged by the intensity value.

**Table 2.2 Detail demographic information of lesion profile.**

| Subject | Age (yrs) | Gender | Chronicity (m) | Stroke type | Group | Affected side | Lesion volume (cm3) | Lesion Location |
| --- | --- | --- | --- | --- | --- | --- | --- | --- |
|  |  |  |  |  |  |  |  |  |
| 1* | 47 | M | 27 | Hemorrhagic | HDtDCS | Left | 29.147 | ALIC, Insula, IFG, Putamen, Postcentral, IPL, MFG |
| 2 | 65 | F | 137 | Ischemic | HDtDCS | Right | 40.367 | ALIC, PLIC, Insula, STG, IPL, Postcentral, Precentral |
| 3 | 41 | F | 70 | Hemorrhagic | HDtDCS | Right | 16.119 | PLIC, Precuneus, STG, OG |
| 4 | 54 | M | 17 | Ischemic | HDtDCS | Right | 0.019 | ALIC |
| 5 | 60 | F | 26 | Ischemic | HDtDCS | Right | 0.307 | Brainstem |
| 6 | 51 | F | 12 | Ischemic | HDtDCS | Right | 0.086 | Putamen, Caudate Nucleus |
| 7 | 50 | F | 16 | Hemorrhagic | HDtDCS | Left | 0.569 | Thalamus, Brainstem |
| 8 | 69 | F | 25 | Ischemic | HDtDCS | Left | 0.323 | Brainstem |
| 9 | 67 | M | 63 | Ischemic | HDtDCS | Left | 1.510 | ALIC, Cingulate Gyrus, Thalamus |
| 10 | 62 | M | 40 | Hemorrhagic | Sham | Right | 0.680 | Putamen |
| 11 | 50 | F | 56 | Ischemic | Sham | Right | 10.543 | ALIC, PLIC, Precentral |
| 12 | 73 | F | 147 | Ischemic | Sham | Right | 7.541 | ALIC, Putamen |
| 13 | 77 | M | 27 | Ischemic | Sham | Left | 31.637 | ALIC, PLIC, Cingulate gyrus |
| 14 | 62 | M | 33 | Ischemic | Sham | Right | 2.682 | Putamen, Claustrum, Insula |
| 15 | 72 | F | 160 | Ischemic | Sham | Left | 4.182 | ALIC, Cingulate gyrus |
| 16 | 59 | M | 16 | Hemorrhagic | Sham | Right | 7.998 | Insula, ALIC, Precentral, Postcentral, Putamen |
| 17 | 44 | F | 28 | Hemorrhagic | Sham | Left | 28.529 | ALIC, PLIC, Precentral, Paracentral Lobule |
| 18 | 53 | M | 32 | Ischemic | Sham | Left | 35.576 | Precuneus, ALIC, Postcentral, Postcentral, Paracentral Lobule, SPL |
| 19 | 69 | F | 83 | Ischemic | Sham | Right | 1.252 | ALIC, Putamen |

*: The subject dropped out after three training sessions because of the COVID-19 epidemic in Hong Kong. The location of lesion were marked using individual structural T1 images by trained professionals. Abbreviations: ALIC=Anterior limb of the internal capsule; F=Female; IPL=Inferior Parietal Lobe; M=Male; MFG=Middle frontal gyrus; PLIC=Posterior limb of the internal capsule; SPL=Superior Parietal Lobe.

**Table 2.3 Clinical scores alteration in two groups.**

| Group | Time | **Clinical scores** | | | | | | | |
| --- | --- | --- | --- | --- | --- | --- | --- | --- | --- |
|  |  | FMAUE | | ARAT | MASw | MASf | MASff |  | MASfe |
| **HDtDCS-group**  **(n = 8)** | Pre | 40.9 ± 6.2 | 30.7 ± 12.2 | | 1.55 ± 1.10 | 1.65 ± 0.98 | 0.50 ± 0.53 |  | 1.15 ± 0.57 |
|  | Post | 48.9 ± 6.5 | 38.6 ± 11.4 | | 0.50 ± 0.76 | 0.62 ± 0.52 | 0.12 ± 0.35 |  | 0.50 ± 0.53 |
|  | Follow up | 49.8 ± 5.7 | 39.5 ± 10.6 | | 0.75 ± 1.04 | 0.60 ± 0.85 | 0.12 ± 0.35 |  | 0.48 ± 0.67 |
| Change | Post - Pre | **8.0 ± 2.5*** | **7.9 ± 1.6*** | | -1.05±0.55* | **-1.02±0.62*** | -0.38±0.52 |  | -0.65±0.67* |
|  | Follow up - Pre | **8.9 ± 2.2*** | **8.8 ± 3.1*** | | -0.80±0.51* | **-1.05±0.55*** | -0.38±0.52 |  | -0.67±0.58* |
| **Sham-group**  **(n = 10)** | Pre | 40.5 ± 11.6 | 29.0 ± 14.5 | | 1.66 ± 0.74 | 1.74 ± 0.92 | 0.50 ± 0.53 |  | 1.24 ± 0.63 |
|  | Post | 45.8 ± 11.5 | 35.5 ± 15.3 | | 1.10 ± 0.57 | 1.32 ± 1.00 | 0.40 ± 0.52 |  | 0.92 ± 0.70 |
|  | Follow up | 45.7 ± 12.1 | 35.1 ± 14.6 | | 1.20 ± 0.63 | 1.28 ± 1.00 | 0.30 ± 0.46 |  | 0.98 ± 0.61 |
| Change | Post - Pre | **5.3 ± 2.4*** | **6.5 ± 2.2*** | | -0.56±0.51* | **-0.42±0.47** | -0.10±0.32 |  | -0.32±0.64 |
|  | Follow up - Pre | **5.2 ± 2.4*** | **6.1 ± 2.6*** | | -0.46±0.41* | **-0.46±0.44*** | -0.20±0.42 |  | -0.26±0.60 |
| Time × Group Interaction Statistics | P value | **0.004** | 0.086 | | 0.167 | **0.038** | 0.327 |  | 0.353 |
|  | Observed power | 0.880 | 0.488 | | 0.364 | 0.628 | - |  | - |
|  | Partial Eta Squared | 0.290 | 0.142 | | 0.106 | 0.185 | - |  | - |
| Intergroup P value | Pa | **0.034** | **-** | | **-** | **0.031** | **-** |  | **-** |
|  | Pb | **0.002** | **-** | | - | **0.023** | **-** |  | **-** |

The interaction P value represents the time × group interaction from the two-way ANOVA results. Pa: Intergroup P value of difference value between post-assessment and baseline, which represents the comparison of the change of parameters at post-assessment between Active-group and Sham-group. Pb: Intergroup P value of difference value between Follow-up assessment and baseline, which represents the comparison of the change of parameters at follow-up assessment between Active-group and Sham-group.

Abbreviations: Active-group, multisite high-definition transcranial direct stimulation with EMG-driven robotic hand group; Sham-group, sham stimulation with EMG-driven robot hand group; FMAUE, Fugl-Meyer motor function assessment of upper extremity; MAS, Modified Ashworth Scale.

**Table 2.4 CMC parameters alteration for HDtDCS-group and Sham-group**

| Group | Time | CMC parameters | |
| --- | --- | --- | --- |
|  |  | ${BetaCMC}_{FDgrasp}$ | ${BetaCMC}_{EDopen}$ |
| **HDtDCS-group**  **(n = 8)** | Pre | 0.071 ± 0.012 | 0.068 ± 0.012 |
|  | Post | 0.086 ± 0.021 | 0.083 ± 0.011 |
|  | Follow up | 0.079 ± 0.010 | 0.084 ± 0.015 |
| Change | Post - Pre | 0.015 ± 0.015 | 0.015±0.007* |
|  | Follow up - Pre | 0.008±0.004* | 0.016±0.011* |
| **Sham-group**  **(n = 10)** | Pre | 0.066 ± 0.010 | 0.067 ± 0.011 |
|  | Post | 0.077 ± 0.009 | 0.080 ± 0.013 |
|  | Follow up | 0.076 ± 0.011 | 0.077 ± 0.011 |
| Change | Post - Pre | 0.011±0.005* | 0.013±0.013 |
|  | Follow up - Pre | 0.010±0.011 | 0.010±0.009* |
| Time × Group Interaction Statistics | P value | 0.553 | 0.415 |
|  | Observed Power | 0.142 | 0.180 |
|  | Partial Eta Squared | 0.036 | 0.118 |
| Intergroup P value | Pa | - | - |
|  | Pb | - | - |

*: The difference value was statistically significant. P < 0.05. The interaction P value represents the time × group interaction from the two-way ANOVA results. Pa: Intergroup P value of difference value between post-assessment and baseline, which represents the comparison of the change of parameters at Post assessment between HDtDCS-group and Sham-group. Pb: Intergroup P value of difference value between Follow-up assessment and baseline, which represents the comparison of the change of parameters at follow-up assessment between HDtDCS-group and Sham-group.

Abbreviations: ${BetaCMC}_{FDgrasp}$, Beta band peak CMC of Flexor digitorum during grasping hand; ${BetaCMC}_{EDopen}$, Beta band peak CMC of Extensor digitorum during opening hand; $Beta{CMC}_{FDopen}$, Beta band peak CMC of Flexor digitorum during opening hand; HDtDCS-group, multisite high-definition transcranial direct stimulation with EMG-driven robotic hand group; Sham-group, sham stimulation with EMG-driven robot hand group.

**Table 2.5 EMG parameters alteration for HDtDCS-group and Sham-group.**

|  |  | ${CI}_{open}$ | ${CI}_{close}$ |
| --- | --- | --- | --- |
| **HDtDCS-group**  **(n = 8)** | Pre | 0.164 ± 0.057 | 0.134 ± 0.057 |
|  | Post | 0.088 ± 0.031 | 0.093 ± 0.049 |
|  | Follow up | 0.067 ± 0.042 | 0.078 ± 0.035 |
| Change | Post - Pre | **-0.076±0.046*** | -0.041±0.069 |
|  | Follow up - Pre | **-0.097**±**0.045*** | -0.056±0.068* |
| **Sham-group**  **(n = 10)** | Pre | 0.129 ± 0.072 | 0.180 ± 0.053 |
|  | Post | 0.107 ± 0.044 | 0.110 ± 0.052 |
|  | Follow up | 0.097 ± 0.056 | 0.106 ± 0.036 |
| Change | Post - Pre | **-0.022±0.037** | -0.070±0.054* |
|  | Follow up - Pre | **-0.032**±**0.070** | -0.074±0.040* |
| Time × Group Interaction Statistics | P value | **0.020** | 0.513 |
|  | Observed Power | 0.718 | 0.155 |
|  | Partial Eta Squared | 0.216 | 0.041 |
| Intergroup P value | Pa | **0.014** | **-** |
|  | Pb | **0.035** | - |

*: The difference value was statistically significant. P < 0.05. The interaction P value represents the time × group interaction from the two-way ANOVA results. Pa: Intergroup P value of difference value between post-assessment and baseline, which represents the comparison of the change of parameters at Post assessment between HDtDCS-group and Sham-group. Pb: Intergroup P value of difference value between Follow-up assessment and baseline, which represents the comparison of the change of parameters at follow-up assessment between HDtDCS-group and Sham-group.

Abbreviations: ${CI}_{open}$, muscle co-contraction index between flexor digitorum and extensor digitorum during opening hand movements; ${CI}_{close}$, muscle co-contraction index between flexor digitorum and extensor digitorum during grasping hand movements

**Table 2.6 Clinical demographic information of HDtDCS-group and Sham-group groups**

| Measures | Pre-intervention HDtDCS-group group (n=8) Mean ± SD | Pre-intervention Sham-group group (n=10) Mean ± SD | P value |
| --- | --- | --- | --- |
| Age (years) | 57.1 ± 9.8 | 62.1 ± 10.8 | 0.33 |
| Chronicity(month) | 45.7 ± 42.9 | 62.2 ± 51.7 | 0.48 |
| Gender (Female/Male) | 6 / 2 | 5 / 5 | 0.46 |
| Affected side (Right/left) | 5 / 3 | 6 / 4 | 0.85 |
| Stroke Type (Ischemia/Hemorrhagic) | 6 / 2 | 7 / 3 | 0.88 |
| Lesion site (Cortico-subcortical*/Subcortical) | 2 / 6 | 5 / 5 | 0.46 |
| Lesion volume (cm^3^) | 7.4 ± 14.4 | 13.1 ± 13.5 | 0.40 |
| FMAUE | 40.9 ± 6.2 | 40.5 ± 11.6 | 0.94 |
| MAS of wrist | 1.55 ± 1.10 | 1.66 ± 0.74 | 0.80 |
| MAS of finger | 1.65 ± 0.98 | 1.74 ± 0.93 | 0.84 |
| ARAT | 30.7 ± 12.2 | 29.0 ±14.5 | 0.79 |

**References**

1. Friston KJ, Williams S, Howard R, Frackowiak RS, Turner R. Movement-related effects in fMRI time-series. Magn Reson Med. 1996 Mar;35(3):346-55. Epub 1996/03/01.

2. Siegel JS, Power JD, Dubis JW, Vogel AC, Church JA, Schlaggar BL, et al. Statistical improvements in functional magnetic resonance imaging analyses produced by censoring high-motion data points. Hum Brain Mapp. 2014 May;35(5):1981-96. Epub 2013/07/19.

3. Thielscher A, Antunes A, Saturnino GB. Field modeling for transcranial magnetic stimulation: A useful tool to understand the physiological effects of TMS? Annu Int Conf IEEE Eng Med Biol Soc. 2015;2015:222-5. Epub 2016/01/07.

4. Gullmar D, Haueisen J, Reichenbach JR. Influence of anisotropic electrical conductivity in white matter tissue on the EEG/MEG forward and inverse solution. A high-resolution whole head simulation study. Neuroimage. 2010 May 15;51(1):145-63. Epub 2010/02/17.

5. Datta A, Baker JM, Bikson M, Fridriksson J. Individualized model predicts brain current flow during transcranial direct-current stimulation treatment in responsive stroke patient. Brain Stimul. 2011 Jul;4(3):169-74. Epub 2011/07/23.

6. Saturnino GB, Madsen KH, Thielscher A. Optimizing the electric field strength in multiple targets for multichannel transcranial electric stimulation. J Neural Eng. 2021 Feb 11;18(1). Epub 2020/11/13.

7. Saturnino GB, Siebner HR, Thielscher A, Madsen KH. Accessibility of cortical regions to focal TES: Dependence on spatial position, safety, and practical constraints. Neuroimage. 2019 Dec;203:116183. Epub 2019/09/17.

8. Hu XL, Tong KY, Wei XJ, Rong W, Susanto EA, Ho SK. The effects of post-stroke upper-limb training with an electromyography (EMG)-driven hand robot. J Electromyogr Kinesiol. 2013 Oct;23(5):1065-74. Epub 2013/08/13.

9. Ramos-Murguialday A, Broetz D, Rea M, Laer L, Yilmaz O, Brasil FL, et al. Brain-machine interface in chronic stroke rehabilitation: a controlled study. Ann Neurol. 2013 Jul;74(1):100-8. Epub 2013/03/16.

10. Bao SC, Wong WW, Leung TWH, Tong KY. Cortico-Muscular Coherence Modulated by High-Definition Transcranial Direct Current Stimulation in People With Chronic Stroke. IEEE Trans Neural Syst Rehabil Eng. 2019 Feb;27(2):304-13. Epub 2019/01/01.

11. Palmer JA, Makeig S, Kreutz-Delgado K, Rao BD, editors. Newton method for the ICA mixture model. 2008 IEEE International Conference on acoustics, speech and signal processing; 2008: IEEE.

12. Winkler I, Brandl S, Horn F, Waldburger E, Allefeld C, Tangermann M. Robust artifactual independent component classification for BCI practitioners. J Neural Eng. 2014 Jun;11(3):035013. Epub 2014/05/20.

13. Kayser J, Tenke CE. Principal components analysis of Laplacian waveforms as a generic method for identifying ERP generator patterns: I. Evaluation with auditory oddball tasks. Clin Neurophysiol. 2006 Feb;117(2):348-68. Epub 2005/12/17.

14. Schoffelen JM, Oostenveld R, Fries P. Neuronal coherence as a mechanism of effective corticospinal interaction. Science. 2005 Apr 1;308(5718):111-3. Epub 2005/04/02.

15. Rossiter HE, Eaves C, Davis E, Boudrias MH, Park CH, Farmer S, et al. Changes in the location of cortico-muscular coherence following stroke. Neuroimage Clin. 2012;2:50-5. Epub 2012/01/01.

16. Frost G, Dowling J, Dyson K, Bar-Or O. Cocontraction in three age groups of children during treadmill locomotion. J Electromyogr Kinesiol. 1997 Sep;7(3):179-86. Epub 1997/09/01.
